# Design, manufacturing, and testing of 3D-printed fittings for ergonomic helmet CPAP devices: a case study

**DOI:** 10.1038/s41598-025-25851-2

**Published:** 2025-11-25

**Authors:** Paweł Płatek, Natalia Daniel, Kamil Cieplak, Marcin Sarzyński, Janusz Kluczyński, Grzelak Krzysztof, Łukasz Wróblewski

**Affiliations:** 1 Faculty of Mechatronics, Armament and Aerospace, Military University of Technology, 2 Gen. S. Kaliskiego Street, 00-908 Warsaw, Poland; 2 Faculty of Mechanical Engineering, Military University of Technology, 2 Gen. S. Kaliskiego Street, 00-908 Warsaw, Poland; 3 2nd Department of Anaesthesiology and Intensive Care, Medical University of Warsaw, Central Teaching Hospital, Warsaw, Poland

**Keywords:** Additive manufacturing, 3D printing, hCPAP, Ergonomic studies, Medical application, COVID-19, Medical research, Biomaterials, Materials for devices, Health care, Disease prevention, Biomedical engineering, Mechanical engineering

## Abstract

This study presents the design, optimisation, and experimental evaluation of additively manufactured fittings for a custom helmet continuous positive airway pressure (hCPAP) system. The project was initiated as a rapid response to the shortage of respiratory support equipment during the COVID-19 pandemic. The inlet and outlet connectors of the helmet were redesigned to comply with the EN ISO 5356–1:2015 standard, reduce mass, and simplify assembly, while ensuring functional reliability and patient comfort. Components were fabricated using fused filament fabrication (FFF) and selective laser sintering (SLS) with medical-grade ABS, TPU-95A, and PA 2200 materials. A comprehensive assessment of material safety was performed, including tests for potential particle detachment under high-flow air conditions. Results confirmed that ABS Medical printed without active cooling exhibited no measurable release of microplastic particles, making it the preferred material for oxygen pathway components. Computational fluid dynamics (CFD) analyses guided the optimisation of the internal diffuser geometry, leading to improved air distribution and reduced perceived noise. The redesigned helmet system was evaluated in clinical trials involving 120 participants, demonstrating good ergonomic performance, low noise levels, and high user comfort. The findings validate additive manufacturing as a practical and reliable method for the small-batch production of medical fittings, particularly under emergency or resource-limited conditions. The proposed design approach highlights the potential of 3D printing to enable flexible, localised, and rapid development of medical devices adapted to current clinical needs.

**Supplementary Information:**

The online version contains supplementary material available at 10.1038/s41598-025-25851-2.

## Introduction

Contemporary additive manufacturing (AM) techniques are gaining interest not only in leading branches of industry^1–3^, science^4^ but also in bioengineering^5–8^ and medicine^5,9–11^. A wide range of 3D printing techniques and a wide spectrum of available materials with diverse physical and mechanical properties enable the rapid production of objects with complex geometric shapes, often impossible to obtain using standard manufacturing technologies^12^. An additional advantage of 3D printing techniques is the elimination of the need for specialized tools, such as injection molds, dies, or matrices, particularly in prototyping and low- to medium-volume production, where conventional tooling would be economically or logistically unjustified. These features make 3D printing techniques particularly attractive for the production of components where the key requirement is rapid manufacturing, dynamic responsiveness to emerging market needs, or the resolution of crises resulting from the unavailability of necessary equipment and spare parts.

One of the exemplary cases demonstrating the potential of AM techniques was problems related to a lack of access to medical equipment during the COVID-19 pandemic, which broke out in 2019^13–19^. In the early stages of the pandemic, due to widespread lockdowns, disrupted logistics chains, and significantly extended delivery times, commonly used 3D printing techniques such as fused filament fabrication (FFF), selective laser sintering (SLS), and stereolithography (SLA) enabled urgent responses to the most pressing needs^20–23^. Using these techniques, it was possible to produce personal protective equipment for medical personnel, various diagnostic equipment and tools that facilitate patient hospitalisation and treatment^24–28^.

Another example indicating the application of 3D printing techniques in supporting the work of medical personnel is the ongoing military conflicts, e.g., in Ukraine or Gaza^6,29,30^. In these situations, 3D printing techniques enable the rapid production of customized parts, and elements of medical equipment that facilitate the work of medical staff in conflict zones, where the delivery of equipment and spare parts can be a prolonged process, sometimes very complicated.

The examples in the literature presented^21,24,25,27,31–35^ demonstrate that currently available low-cost desktop 3D printers, combined with a wide range of materials for biomedical use, provide an attractive alternative to address urgent crises in healthcare arising from shortages of medical equipment and supplies. Additionally, these 3D printers allow rapid adaptation of the manufactured range of products, modification of their functional features based on needs, and implementation of significant design changes according to the operational requirements of the used equipment.

Since the 1990s, hCPAP has been recognized as an effective method for managing acute respiratory failure^36,37^. It operates by delivering through the helmet a continuous positive pressure, which is higher than atmospheric pressure, along with a specified oxygen fraction to the airways and lungs during both inspiration and expiration. The primary advantage of hCPAP in the treatment of acute hypoxemic respiratory failure (AHRF) is that it eliminates the need for tracheal intubation, sedation, and mechanical ventilation. Furthermore, the helmet interface, compared to other types of interface, offers effective isolation for infected patients, significantly reducing the risk of contamination and infection transmission with better toleration for long-term use (2–3 weeks) than the face mask for CPAP.

**Fig. 1:**
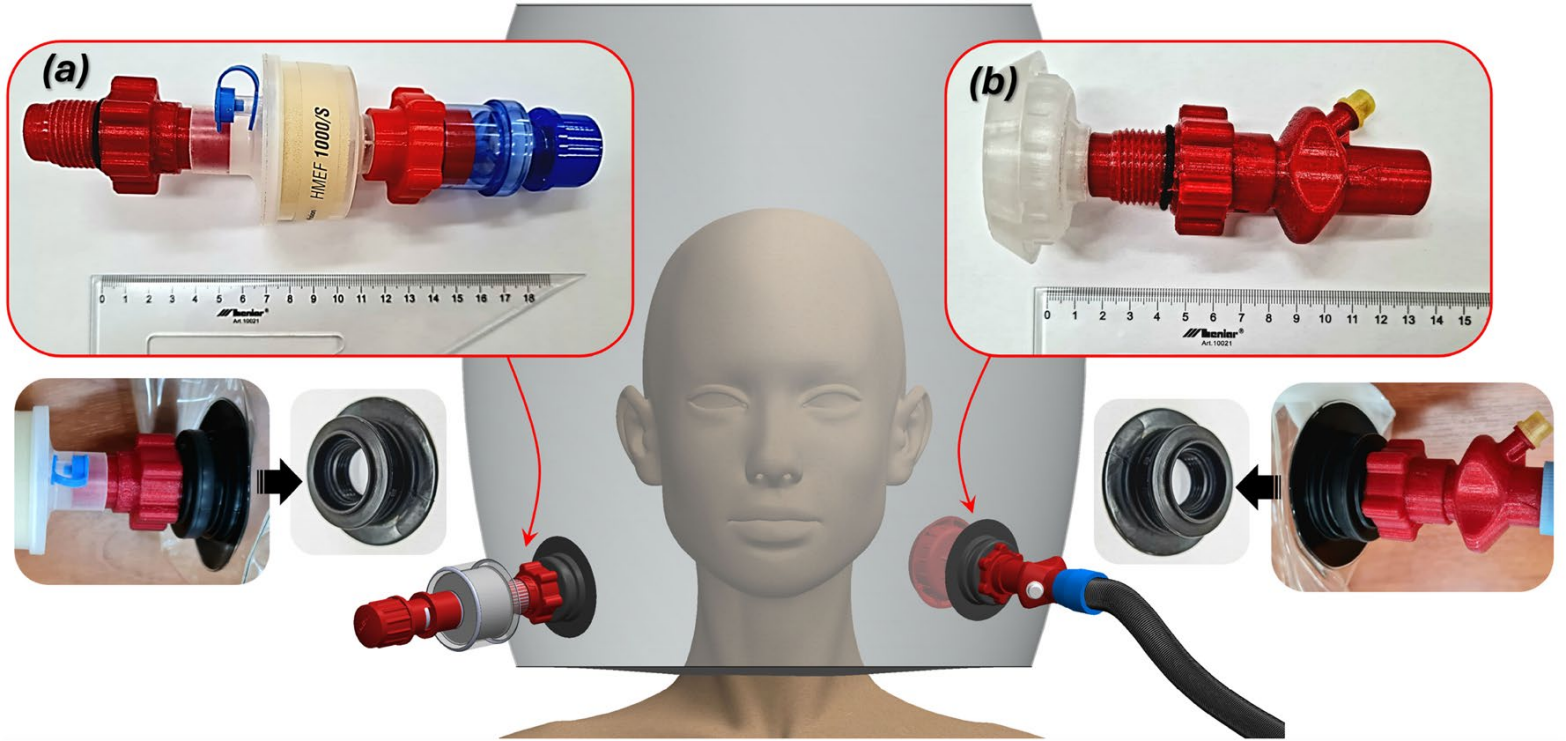
The main view of the preliminary concept of the hCPAP helmet designed and manufactured by the Authors as a project initiative in the fight against COVID-19: (**a**) outlet connector components: the HEPA filter and the PEEP valve, (**b**) inlet connector components: the internal diffuser and the port for oxygen supply.

In Fig. 1, a general view of the preliminary concept for helmet design proposed by the Authors is shown, together with the input (Fig. 1a) and outlet (Fig. 1b) connectors, designed and manufactured using 3D printing technology. The hCPAP helmet depicted in the figure, described in the^16^ consists of a polyurethane helmet, to which standard threaded sockets (known as Boston valves) were attached via vibration welding.

The outlet connector (Fig. 1a), sealed with an O-ring, integrates a HEPA filter and a PEEP valve to regulate the internal pressure of the helmet. The inlet connector features (Fig. 1b) an internal diffuser to direct airflow and includes a port for oxygen supply. The initial designs were found to be oversized and required refinement to reduce the dimensions of the connector and eliminate threaded connections.

This study aims to present the experiences and results of a project initiative carried out by a multidisciplinary research team, which, during the COVID-19 pandemic, developed, manufactured and temporarily deployed a custom-designed helmet continuous positive airway pressure (hCPAP) medical device (a helmet with connection fittings) to treat acute respiratory failure resulting from COVID-19^16^.

The purpose of this study is to demonstrate the potential of additive manufacturing as an interesting and promising method for the small-batch production of components used in testing and research related to the development of new products. This approach is also presented as a viable solution in situations where conventional manufacturing techniques are limited or unjustified due to low production volumes. An additional objective of the study was to show the design process of helmet fitting components, adapted to the technological requirements of 3D printing. The freedom of geometric design, combined with the ability to use materials with various mechanical properties, such as hyper-elastic TPU-95, allowed the authors to achieve the desired functionality straightforwardly. The main idea was to highlight 3D printing as a versatile manufacturing method that can be successfully applied to the production of a wide range of medical equipment, particularly intended to support healthcare in challenging conditions, such as those present in conflict zones.

This study presents the optimization of the original connector design, aiming to minimize mass, simplify geometry, remove threaded joints and O-ring seals, and adapt both connectors for 3D printing. While the COVID-19 related equipment shortage has since been resolved, the study offers valuable design guidelines, key engineering solutions, and insights into the challenges encountered during the 3D printing of medical fitting components.

## Geometrical and functional optimization of hCPAP connectors

**Fig. 2:**
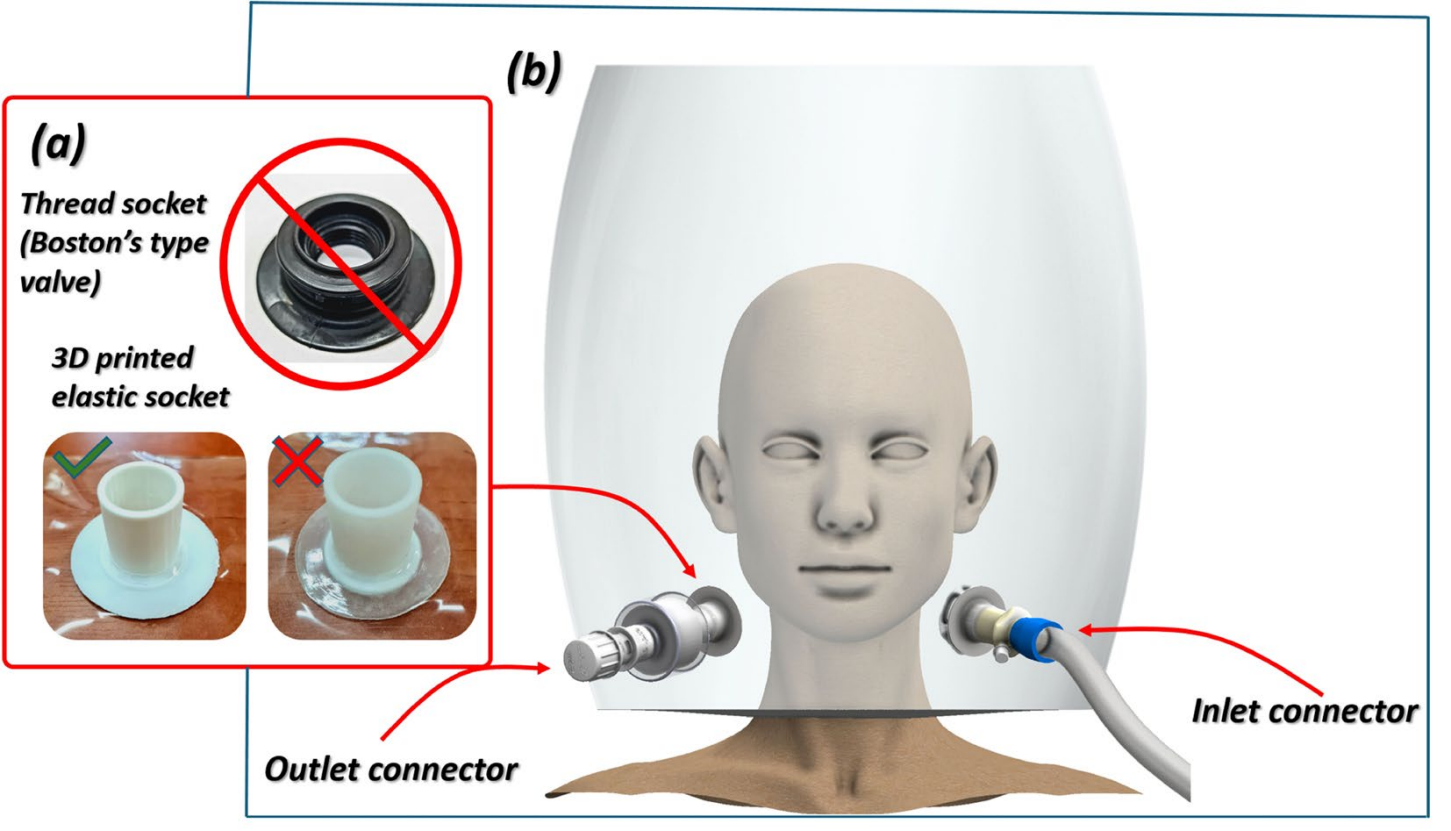
The main view of the modified version of the hCPAP design concept: (**a**) proposed solution of elastic sockets, (**b**) – main view of the new design concept of inlet and outlet connectors.

Developing an optimal geometric solution for helmet connectors required the elimination of the previously used threaded sockets, which affected the dimensions and weight of the individual components. Based on a literature review, it was determined that the geometry of the connecting elements should comply with the standard specified in EN ISO 5356–1:2015, which precisely defines the shape and dimensions of the connectors used in anesthesia and respiratory equipment^38^. Taking into account the guidelines on the shape and dimensions of the connectors and sockets, it was decided that the connector would be welded to the helmet using press-fit fittings (Fig. 2). Before starting the design work, an additional test was performed that involved the production of cylindrical samples made of TPU material (Fig. 2a). These samples were then subjected to an attempt to bond to the material (transparent TPU approved for skin contact) used for helmet production, using vibrational welding. The tested sockets were produced in two variants: the first using the FFF 3D printing technique with PolyFlex TPU-95 material (PolyMaker) and the other using the SLS 3D printing technique with TPU 1301 material (EOS GmbH). Despite positive results on the connection quality between the TPU 1301 material and the helmet material, it was decided that the SLS technique should not be used to produce components of the inlet assembly. Unsintered and loose particles of the feedstock material, in the form of powder (ranging from 22 to 138 µm), could enter the respiratory system of patients undergoing oxygen therapy through the inlet connection, posing a serious health risk^39–41^. In the case of the FFF technique, no material structures that could be detached from the internal walls due to high airflow were observed. Therefore, it was decided that the inlet and outlet socket connectors would ultimately be manufactured from PolyFlex TPU 95 material. The vibration welding process was used specifically to join the printed T22F socket, made of TPU-95, to the corresponding cutout in the helmet shell, which was also fabricated from thermoplastic polyurethane. The compatibility of both materials allowed local melting at the interface, ensuring a uniform, airtight, and mechanically durable bond. This assembly approach eliminated the need for chemical adhesives and preserved the biocompatibility of the overall system.

**Fig. 3:**
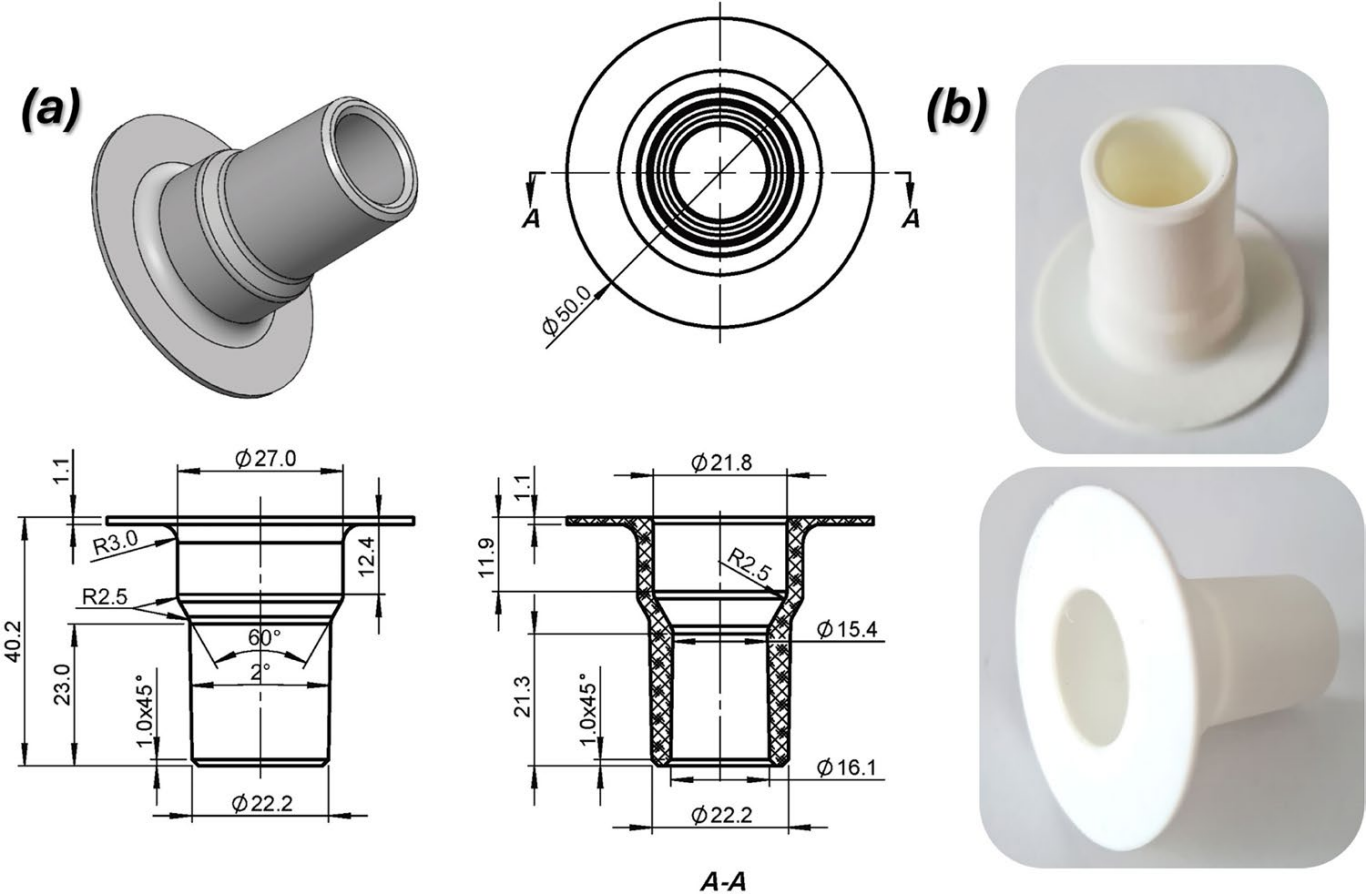
The main view of the helmet socket designed according to the requirements defined in EN ISO 5356–1:2015 (size ‘22’) (**a**)—technical draw, (**b**) – 3D printed socket using the FFF technique with the use of PolyFlex TPU 95 material.

According to the EN ISO 5356–1:2015 guidelines, the connectors for the system were designed in the standard “22” size. To streamline the manufacturing process and standardize the components, a unified socket geometry was developed for the inlet and outlet assemblies of the helmet. Figure 3 provides detailed specifications of the shape and dimensions, which are designed to ensure compatibility between the hCPAP device and the respirator. By adapting both the input and outlet assemblies to the standardized geometry, the system achieves enhanced interoperability and eliminates the need for additional intermediary components.

To ensure proper press fit functionality, the helmet sockets were designed in accordance with EN ISO 5356–1:2015, which defines dimensional tolerances for 22 mm conical connectors commonly used in anesthetic and respiratory equipment (Fig. 3a). While this standard served as the design basis, additive manufacturing, specifically FFF, presents known limitations in terms of geometric accuracy, surface quality, and repeatability. Consequently, several iterations of the connector were printed and manually evaluated to verify fit, mechanical integrity, and leak resistance (Fig. 3b). The TPU-95 material was selected for its Shore A hardness of 95, high elongation, and elastic recovery, which allowed it to form a secure yet removable connection with rigid elements of the breathing system. These mechanical characteristics compensate for dimensional deviations that may result from the AM process and eliminate the need for additional sealing elements such as O-rings or adhesives.

The key stages of the described process, including the detailed geometry design, material selection, and development of the component joining technique, are presented in detail in the following subsections.

### Geometrical and functional optimization of inlet and outlet connectors

**Fig. 4:**
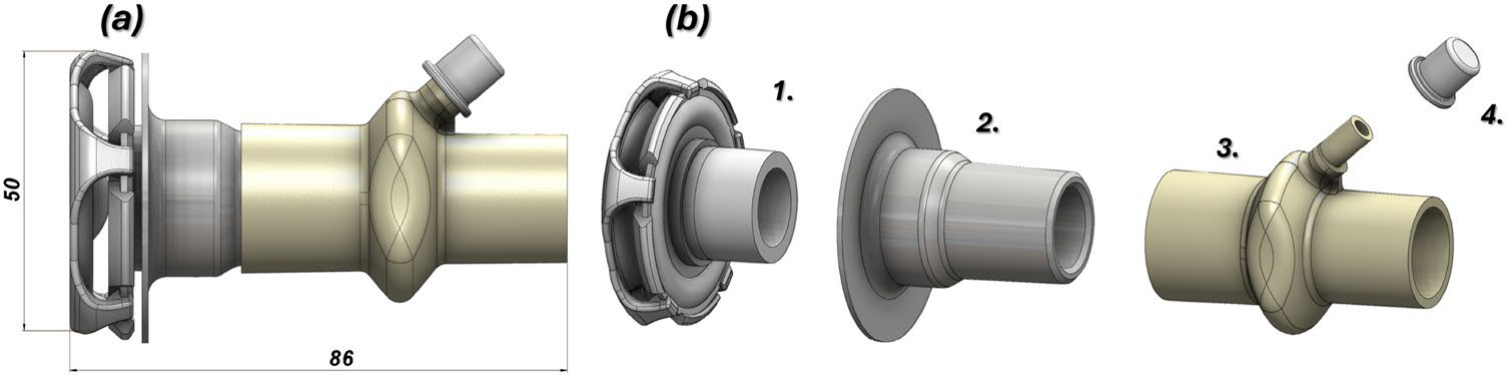
The main view of the 3D CAD model of inlet connector: (**a**) assembled with overall dimensions, (**b**) in exploded view, where: 1 – diffuser, 2) – flexible socket of type T 22F-type, 3) – connector with additional oxygen port, 4) – cap.

The next stage in completing the design task within the CAD system involved optimizing the geometry of the inlet and outlet connectors. Design work was carried out using the SolidWorks 2019 software. A detailed characterization of the air delivery subsystem for the helmet is presented in Fig. 4. It consists of a a diffuser (1), described previously flexible T 22F type socket (2), an inlet connector with oxygen port (3) and cap (4).

A key aspect of this process involved CFD simulation studies aimed at enhancing the diffuser’s efficiency and reducing its overall dimensions, as proposed in the preliminary solution illustrated in Fig. 1b. The original concept, described in^16^, had not been optimized to achieve minimal size and weight. During oxygen therapy, the diffuser serves two critical functions: improving patient comfort by mitigating the direct impact of strong airflow on the face and enhancing air circulation to reduce carbon dioxide accumulation within the helmet. Preliminary CFD analyses of the initial diffuser concept revealed that the proposed solution was suboptimal. Airflow was excessively dispersed against the helmet walls, potentially impeding effective removal of carbon dioxide. Additionally, the initially proposed one-way valve design required modifications to eliminate the spring and reduce weight^16^.

In medical applications, several types of one-way valves are employed to prevent backflow of gases or liquids, including spring-loaded needle valves, ball check valves, and flap or disc valves. Among these, membrane-based designs, particularly umbrella valves, are widely adopted due to their simplicity, compact geometry, and reliable operation under low-pressure differentials typical for respiratory and fluid delivery systems. The umbrella valve was selected in this study because it provides consistent unidirectional flow control, and it can be easily integrated into thermoplastic components. The high flexibility of medical-grade silicone which is used in umbrella valve production, ensures effective prevention of reverse airflow during patient exhalation. This updated solution aligns with the objectives of reducing both weight and complexity while maintaining high functional reliability.

**Fig. 5:**
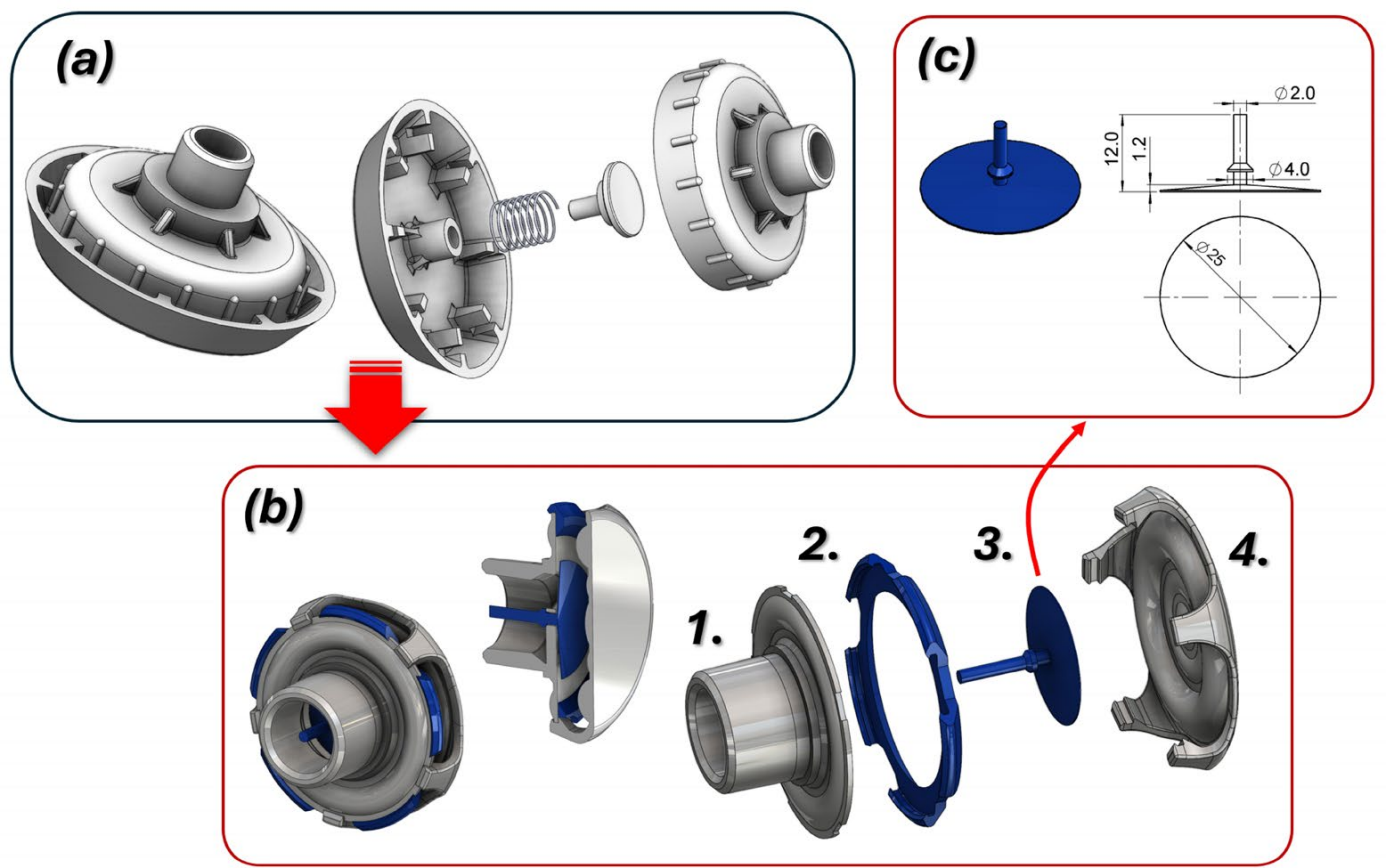
The main view of the diffuser design concept: (**a**) – preliminary shape and main dimensions, (**b**)—geometry after CFD studies: 1 -connector housing, 2 – flow guide ring, 3 – umbrella valve, 4 – outlet cap, c) – geometrical features of umbrella valve.

Figure 5 illustrates a general view of the preliminary and modified design concepts of the diffuser that meet the design and technological requirements. The component comprises three parts, with geometry tailored to the specific demands of the FFF 3D printing process. As an optional feature, provision has been made for the installation of an additional umbrella valve. The shapes of individual components were designed to eliminate the need for support material during the 3D printing process.

The assembly of diffuser parts is facilitated through snap-fit connections, ensuring simplicity and reliability. A ring-shaped element inside the cover has been incorporated to direct airflow towards the patient’s face, improving therapeutic efficiency. The geometry of the mounting fitting has been designed to align with the dimensions of flexible connectors, proposed to be manufactured using TPU-95 material via 3D printing.

**Fig. 6:**
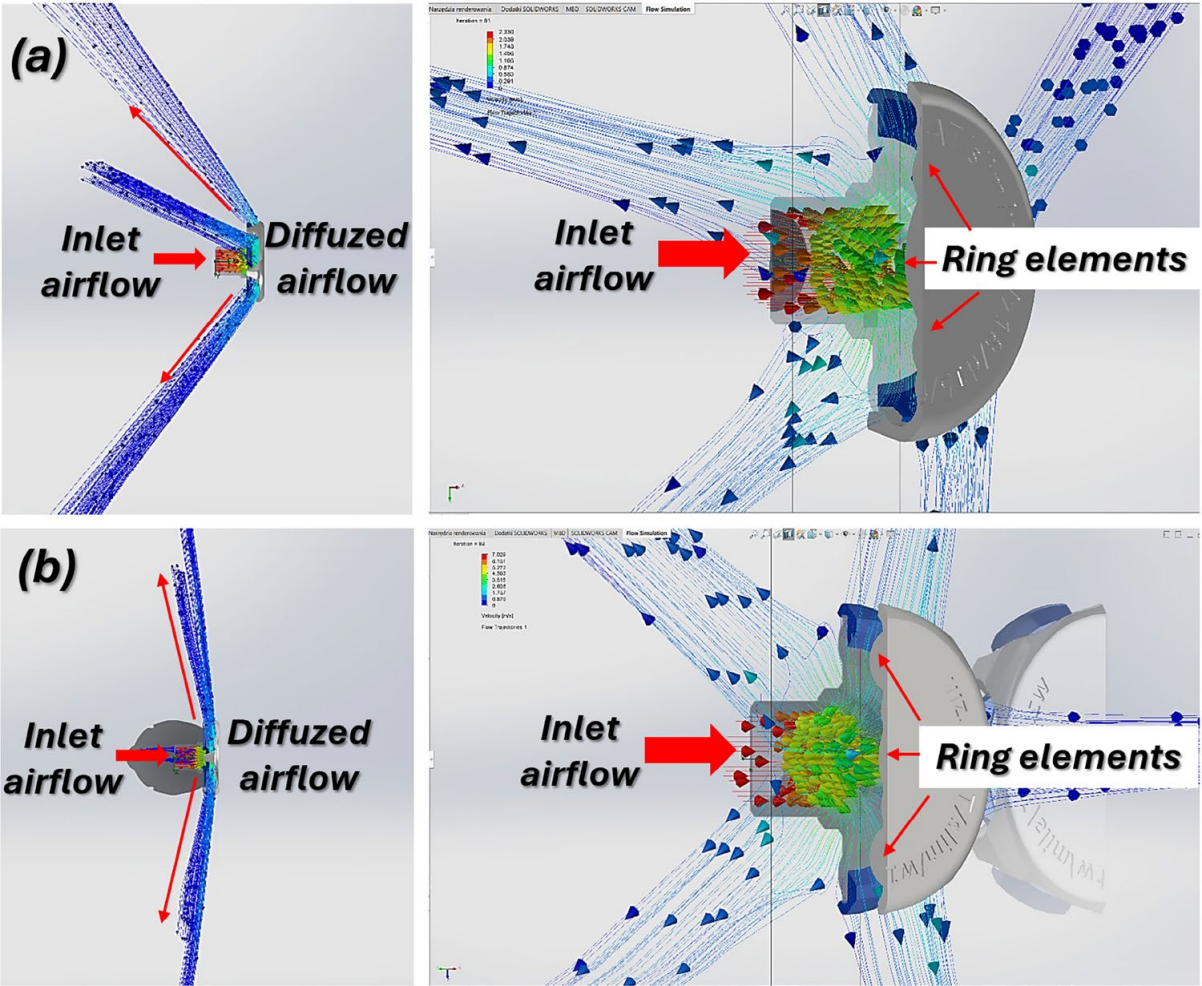
Visualization of CFD studies illustrating the airflow direction from the diffuser without valve: (**a**)—flow rate 20 l/min, (**b**)—flow rate 60 l/min.

**Fig. 7:**
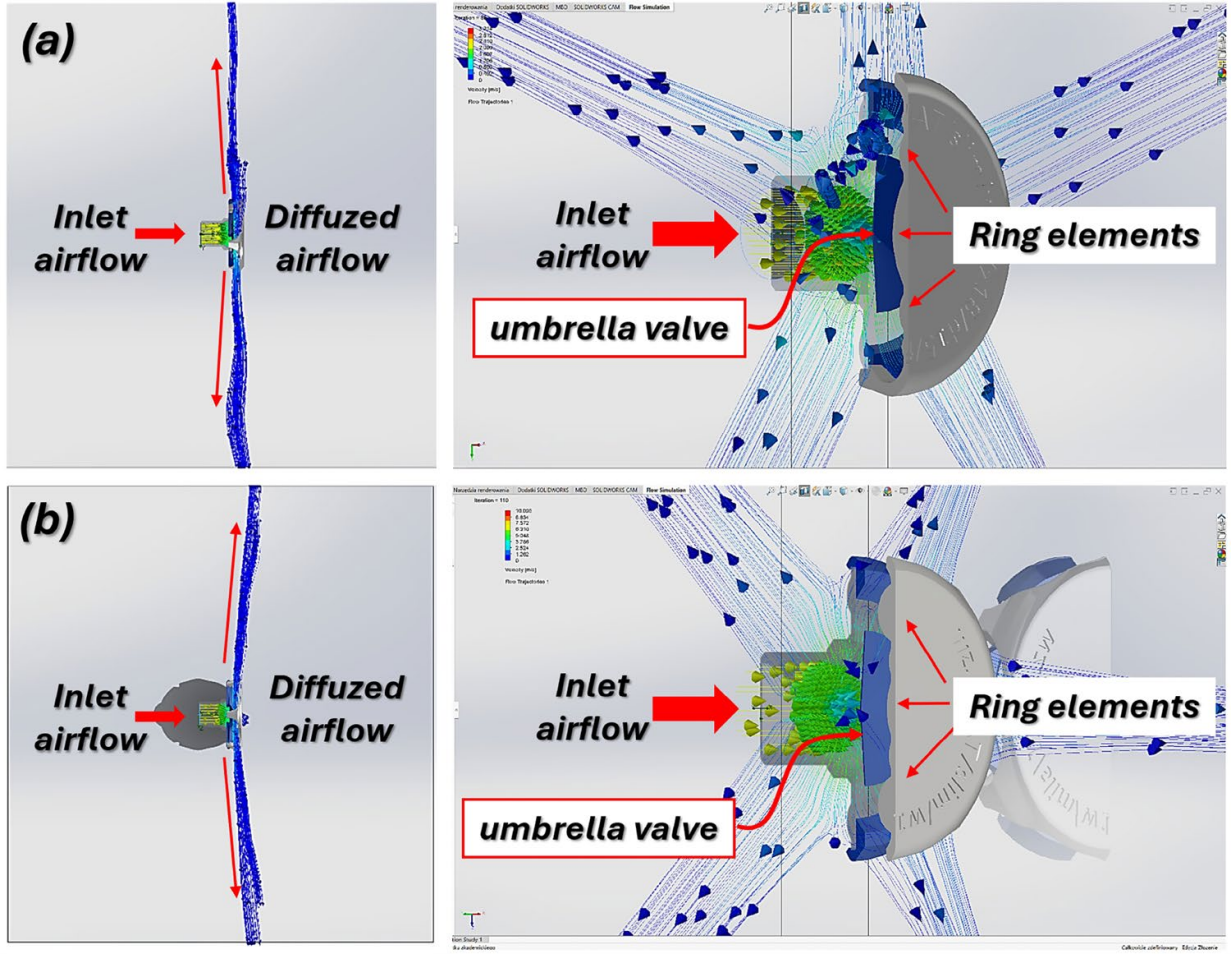
Visualization of CFD studies illustrating the airflow direction from the diffuser with umbrella valve: (**a**)—flow rate 20 l/min, (**b**)—flow rate 60 l/min.

The compact size and rounded edges prevent discomfort for patients and interference with medical personnel during helmet application or removal. Its design minimizes the risk of damaging either the diffuser or the polyurethane helmet. The CFD simulations, conducted using the CAE module in SolidWorks 2019, evaluated performance changes from geometric modifications at airflow rates of 20 l/min and 60 l / min, common in clinical settings. Two configurations were tested: without and with an umbrella valve. Without the valve, the airflow is directed by a circular profile on the inner diffuser cover, crucial to guide the stream (Fig. 6). With the valve, this guiding role is reduced (Fig. 7). The ring-shaped element on the diffuser cover significantly influences the volume of forward-directed airflow, essential for maintaining air circulation within the helmet, ensuring both functionality and patient comfort. Figures 6,  7 illustrate the airflow distribution generated by the diffuser in the configurations without and with the umbrella valve at flow rates of 20 l/min and 60 l/min.

The presented airflow analysis clearly demonstrates the advantages of incorporating an umbrella valve into the diffuser design (Fig. 7). In the configuration with the valve, the incoming airflow is redirected laterally and dispersed through the surrounding ring elements, preventing a concentrated jet from being projected directly towards the patient’s face. This redirection of the flow not only ensures a more uniform distribution of air within the helmet but also significantly enhances patient comfort by reducing the sensation of direct airflow impact. In contrast, the variant without the umbrella valve allows the incoming jet to propagate forward with less deflection, which may cause discomfort during prolonged therapy. Consequently, the integration of the umbrella valve provides a more favourable balance between effective air diffusion and patient well-being, making it a more suitable solution for clinical use in non-invasive respiratory support.

**Fig. 8:**
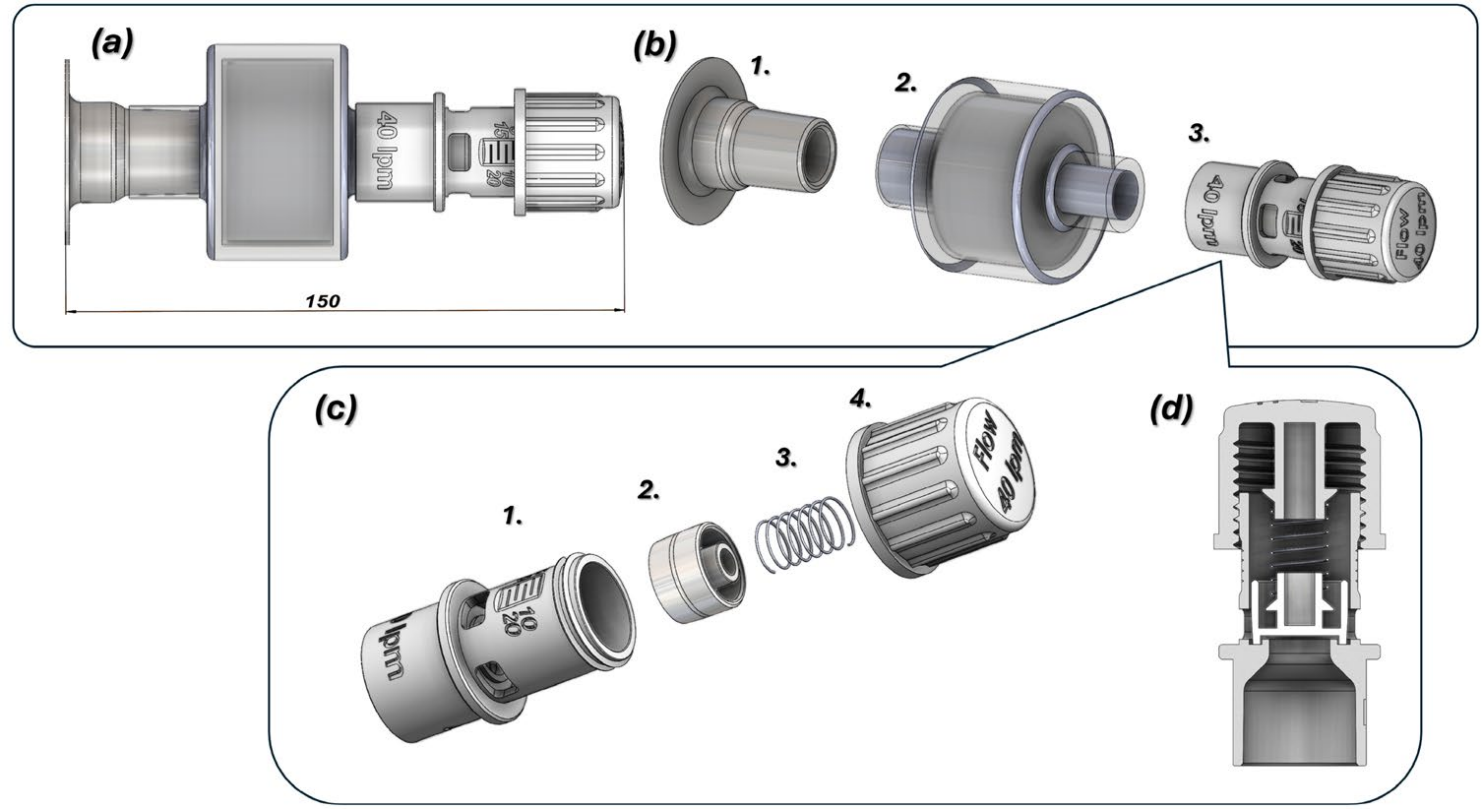
The main view of the 3D CAD model of outlet connector: (**a**) – assembled with overall dimension, (**b**) in exploded view, where: 1) – flexible T 22F-type socket, 2) – HEPA filter, 3) – PEEP valve, (**c**)—exploded view of the PEEP valve, where: 1) – body of PEEP valve, 2) – piston, 3) – spring, 4) – cap nut, d)—cross-section view of the PEEP valve.

The next stage of the project focused on revising the geometry of the 3D CAD models for components of the helmet’s outlet connector. The preliminary concept is shown in Fig. 1a, while the final geometry, presented in Fig. 8. It was developed to comply with EN ISO 5356–1:2004, thereby enabling the use of standard HEPA filters. We then carried out manufacturing trials to assess the feasibility of producing the PEEP valve by Additive Manufacturing Fused Filament Fabrication (FFF) and Selective Laser Sintering (SLS). On the basis of these trials, we defined optimal process parameters that allowed reliable fabrication of the final valve design using both SLS and FFF.

The PEEP valve is a key component that allows the discharge of exhaled carbon dioxide from the helmet while maintaining adjustable air pressure levels during oxygen therapy, depending on the patient’s condition. It regulates pressure within the helmet in the range of 5–20 cm H₂O. Preliminary design and manufacturing tests using additive manufacturing techniques (FFF and SLS) confirmed the feasibility of 3D printing for valve production. The initial designs featured a spring-supported piston, with springs made of stainless steel or 3D-printed polymer. However, the polymer springs showed inconsistent performance under variable temperature and humidity conditions. Additionally, the use of a large thread pitch led to excessive spring tension with minimal angular rotation of the valve cap, limiting pressure regulation. The tests also revealed the need to rescale the valve cap in the 0XY plane to ensure smooth rotation.

Based on these findings, several objectives for an optimal design:

- Application of a stainless steel spring-supported piston;
- Using a fine-pitch thread to provide a wide range of pressure regulation;
- Minimizing the number of components;
- Design of valve body and cap geometry to avoid post-processing and the use of support structures during 3D printing;
- Reduce the production time per valve.

These requirements were fully implemented in the final PEEP valve design, developed using CAD tools. The resulting geometry meets all the technical and manufacturing constraints. A general view of the valve is shown in Fig. 8c and d. It consists of four parts: (1) the body, (2) the piston, (3) the spring, and (4) the cap nut.

**Fig. 9:**
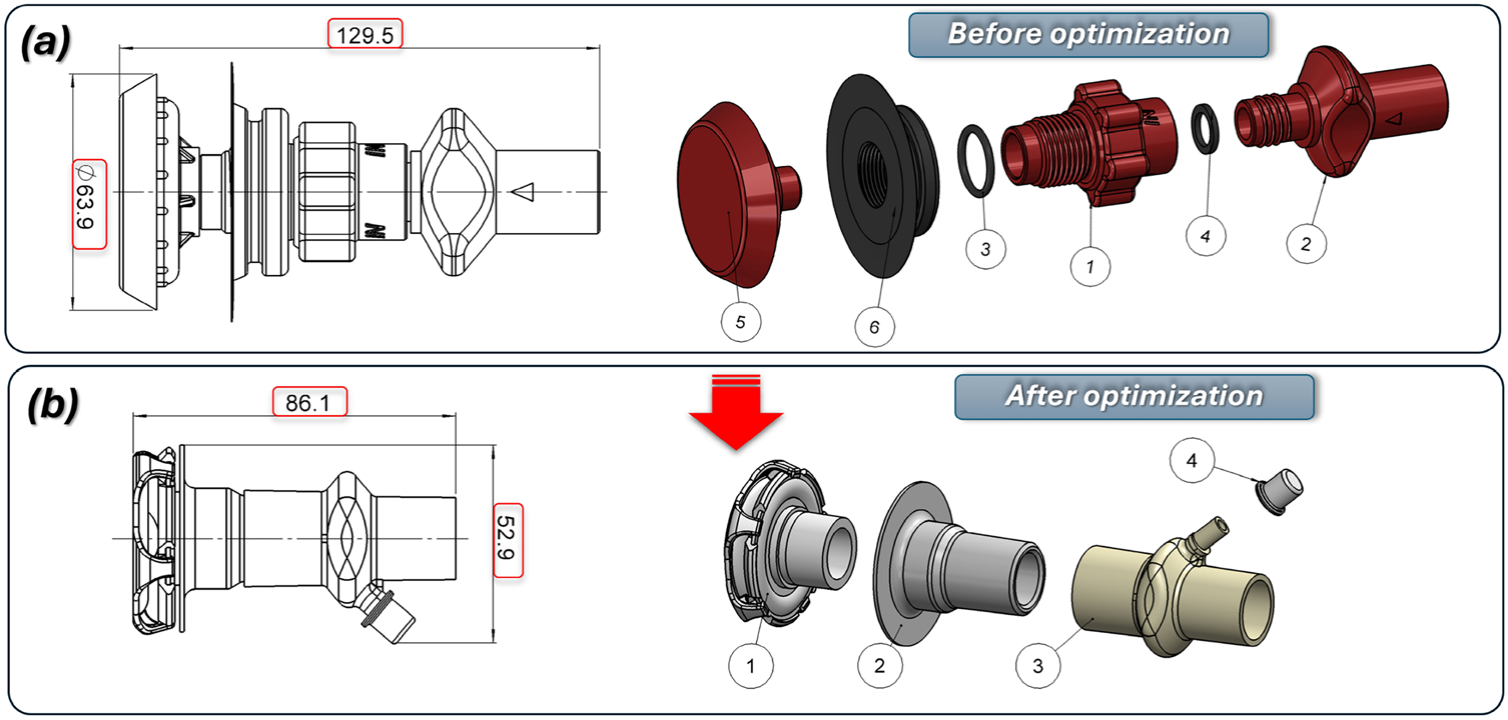
The main view of modification proposed to reduce the size and mass of the inlet connector assembly: (**a**) – before: 1 – main connector body, 2 – outlet part, 3,4 – O-ring seals, 5 – diffuser, 6 – boston type socket, (**b**) – after redesign process: 1 – diffuser, 2 – flexible T 22F type socket, 3 – connector with an additional oxygen port, 4 – cap.

**Fig. 10:**
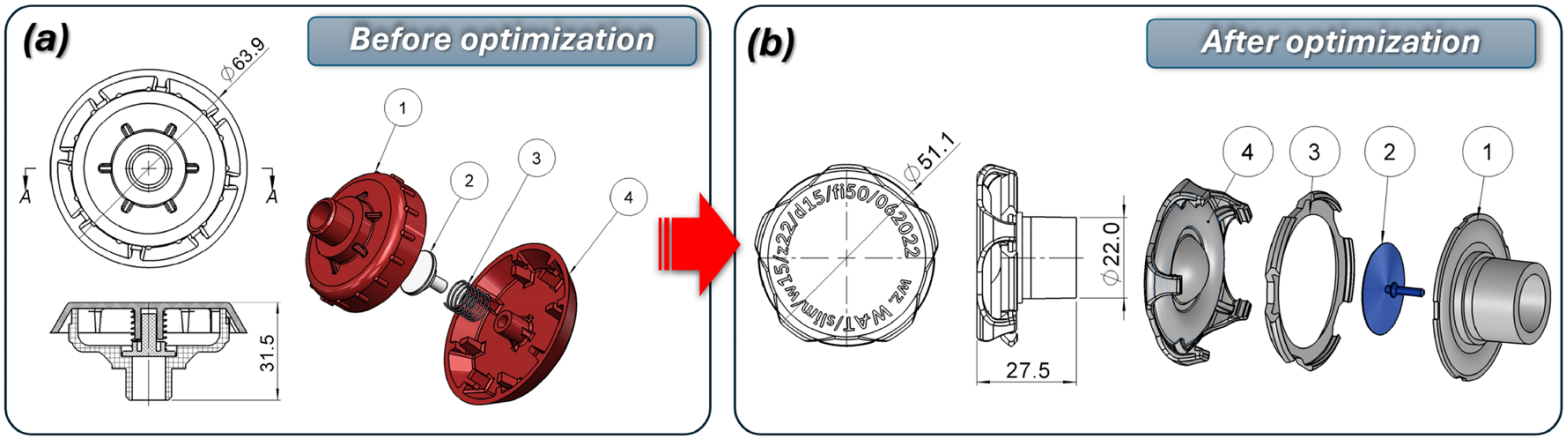
The main view of the modification proposed to reduce the size and mass of the diffuser assembly: (**a**) before, (**b**) after redesign process.

**Fig. 11:**
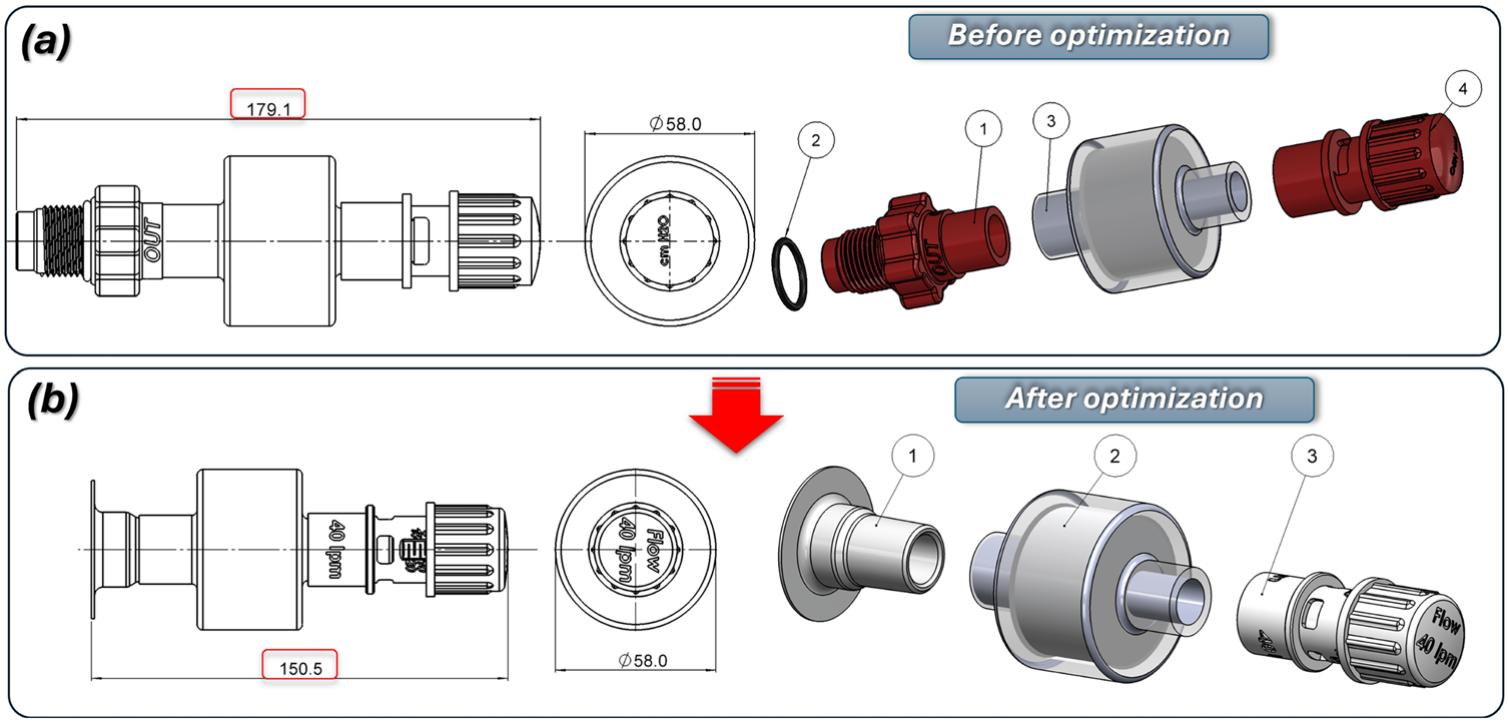
The main view of modification proposed to reduce the size and mass of the outlet connector assembly: (**a**) before: 1 – outlet connector, body, 2 – O-ring seal, 3 – HEPA filter, 4 – PEEP valve (**b**) after redesign process: 1 – flexible T 22F type socket, 2 – HEPA filter, 3 – PEEP valve.

To ensure a proper interface between the valve and the HEPA filter, the valve body fitting was designed in accordance with EN ISO 5356-1standard. To provide an appropriate range of piston-spring adjustment, an M24 × 2.5 thread was adopted. Flow-rate guidance is engraved on the side of the valve body and on the cap face, and a machined flat surface on the body allows a pressure scale to be applied. The valve was calibrated experimentally. Four rectangular outlet ports on the body permit free airflow from the valve. Redesigning the connecting fittings between the CPAP helmet and the ventilator reduced overall size and mass, simplified the connection method, and aligned the parts with additive-manufacturing constraints. The Boston connectors shown in Fig. 1 were replaced with dedicated 22F taper connectors conforming to EN ISO 5356-1, which streamlined the original layout. The new connector, produced by FFF using flexible TPU-95A PolyFlex, replaces the previous threaded joint with a press-fit. This change reduces the envelope of both the inlet and outlet connector assemblies and lowers the part count. Eliminating the thread also removes the need for additional seals, reducing the risk of leakage from the helmet. Figures 9, 10, 11 present a comparison of the 3D CAD models, highlighting the overall dimensions of the components before and after the proposed modification. An additional advantage of the modified geometry of both connector variants was the reduction in their mass.

The comparison presented in Fig. 9 highlights a significant modification of the inlet connector assembly. The overall length has been reduced from 129.5 to 86.1 mm, while the maximum outer diameter has been reduced from Ø 63.9 to 52.9 mm. The component count was lowered from six in the baseline variant (including two O-rings and a Boston-type socket) to four after redesign, owing to the integration of the sealing elements and socket into a flexible T-22F interface. This modification eliminates two sealing joints and one threaded coupling, thereby shortening the airflow path and reducing potential leakage points, assembly time, and tolerance-stack issues. The diffuser element has been retained but located closer to the helmet interface. Together with the reduced weight and shorter overhang, this adjustment is expected to decrease bending moments acting on the helmet shell and improve patient comfort. Furthermore, the redesigned connector incorporates an additional oxygen port directly within the main body, which removes the need for an external adaptor and simplifies hose routing. Overall, the new configuration is more compact, lighter, and easier to assemble and sterilise. The use of a flexible socket improves mechanical compliance, enhancing connection stability and reducing the risk of accidental decoupling. An additional factor contributing to the reduction in the dimensions and mass of the inlet subassembly was the implementation of a redesigned diffuser. Figure 10 presents a comparison of the 3D CAD models of both diffuser variants, along with the corresponding values of their key overall dimensions.

The original diffuser assembly is shown in Fig. 10a, consisting of body components (1), (2), a spring (3), and a cover (4). The redesigned assembly in Fig. 10b consists of the main diffuser body (1), the integrated umbrella valve (2), a retaining clip (3) and the front cover (4). This new design features significantly smaller overall dimensions, while also demonstrating enhanced operational efficiency, as validated through computational fluid dynamics (CFD) analysis. Furthermore, the integration of an umbrella valve serves as a critical improvement, enabling one-way flow control. This prevents air from escaping from the helmet into the inlet system and the connected respirator, ensuring optimal performance and safety. The redesigned diffuser significantly improved patient comfort. CFD-guided modifications to the housing geometry optimized the internal ring shape, redirecting airflow laterally for better distribution. The symmetrical positioning of the ports and mounting sockets in the lower section enhanced comfort during prolonged therapy. A medical-grade silicone umbrella valve was integrated into the diffuser, preventing the backflow of exhaled air into medical devices, thus increasing the safety and efficiency.

The original outlet connector is shown in Fig. 11a, which consisted of an inlet body part (1), an O-ring seal (2), an outlet body part (3), and an adjustment element (4). Following the redesign, the mass was decreased from 120 to 34 g. The final form shown in Fig. 11b was developed to adapt the PEEP valve body to the EN ISO 5356-1:2004 standard, which enables the use of standard HEPA filters. This optimized assembly consists of a flexible T 22F-type socket (1), a HEPA filter (2), and a PEEP valve (3). The PEEP valve is a key component that allows the discharge of exhaled carbon dioxide from the helmet while maintaining adjustable air pressure levels.

The authors optimisation of helmet inlet and outlet connectors geometry aimed to support Additive Manufacturing (AM), allowing rapid, tool-free production of fixture sets required for clinical trials. Small-batch fabrication allowed continuous design improvements based on experimental feedback, eliminating early-stage flaws. Iterative adjustments ensured a secure fit between the fittings, the helmet, and the oxygen therapy devices. To ensure a proper press-fit between the connector components and the helmet sockets, all elements were produced using nominal dimensions based on CAD models, without post-processing. This was made possible because of the intrinsic elasticity of the ABS Medical material used in the fabrication process. Furthermore, to avoid potential dimensional discrepancies between parts made on different machines, all mating elements of a single component set were manufactured on the same FFF 3D printer. This approach guaranteed consistency of dimensions and assembly tolerances and ensured a smooth fit without the need for adhesives or machining. The geometry of the snap-fit joints was refined iteratively during prototyping, allowing secure assembly while avoiding stress concentrations or damage to the press-fit features.

Geometric refinement of adjustable components, such as the PEEP valve regulator cap, allowed precise fitting and consistent pressure regulation. Initial production of inlet and outlet connectors was performed using PET-G and ABS Medical with 3D FFF printing. To assess the risk of particle detachment under high airflow, tests were carried out that confirmed that ABS Medical showed superior resistance to material erosion. Due to ABS’s sensitivity to thermal shrinkage, shielding was applied during printing to reduce air circulation and distortion.

All components in the inlet connector were designed within FFF constraints. They were optimized to eliminate the need for support structures, reducing print time, material waste, and enhancing production efficiency. To clarify the relationship between individual parts, their manufacturing techniques, materials, and functional justification, Table 1 summarizes all key components used in the hCPAP system. The figures illustrating the 3D-printing process for the individual components of the hCPAP helmet fittings are included in the Appendix to this manuscript.

**Table 1:** Summary of the inlet and outlet connector components, materials used, and rationale for selection.

Component (Figure no.)	Manufacturing method	Material used	Justification for material selection
T22F-type socket (Fig. A1)	FFF (Prusa i3 MK3S +)	TPU-95 PolyFlex	Flexible, biocompatible, enables vibration welding with helmet shell, press-fit compatible with tubing
Inlet connector with oxygen drain (Fig. A2)	FFF (Prusa i3 MK3S +)	ABS Medical	Biocompatible (USP VI, ISO 10,993–1), stable under airflow, strong mechanical properties
Cap for oxygen drain (plug)	FFF (Prusa i3 MK3S +)	TPU-95 PolyFlex	Elasticity ensures tight sealing; compatible with small-diameter oxygen drain
Diffuser (internal inlet) (Fig. A3)	FFF (Prusa i3 MK3S +)	ABS Medical	Modular, no supports needed, good CFD performance, stable under sterilisation
PEEP valve (Fig. A4)	SLS (Formiga P110)	PA 2200 (Polyamide)	Durable, safe for external outlet, no internal flow contact
Spring (inside PEEP valve)	–	Stainless Steel	Consistent elasticity under clinical conditions, reliable repeatability
Umbrella valve (not shown)	–	Medical-grade Silicone	Ensures one-way flow, prevents backflow of exhaled air, improves patient safety

SLS 3D printing is viable for external PEEP valve parts, where airflow exposure is not a concern. Additional design modifications further streamlined production by enabling direct printing with minimal post-processing.

## Materials and methods

Functional test components were manufactured using the FFF 3D printing method on a Prusa i3 MK3S + printer (Prusa Research, Czech Republic) with medical-grade ABS (Spectrum Filaments, Poland), PET-G (Spectrum Filaments, Poland), and TPU-95 (Polymaker, China) filaments. The G-codes were generated using Prusa Slicer software. PEEP pressure valve components were produced using the SLS method with a Formiga P110 printer (EOS GmbH, Germany) and medical-grade PA 2200 polyamide. As part of the study, additional experimental tests were conducted to determine whether high-flow air passing through the inlet connector could cause material particles to erode from the internal walls.

### Assessment of airflow-induced erosion in 3D-printed parts

**Fig. 12:**
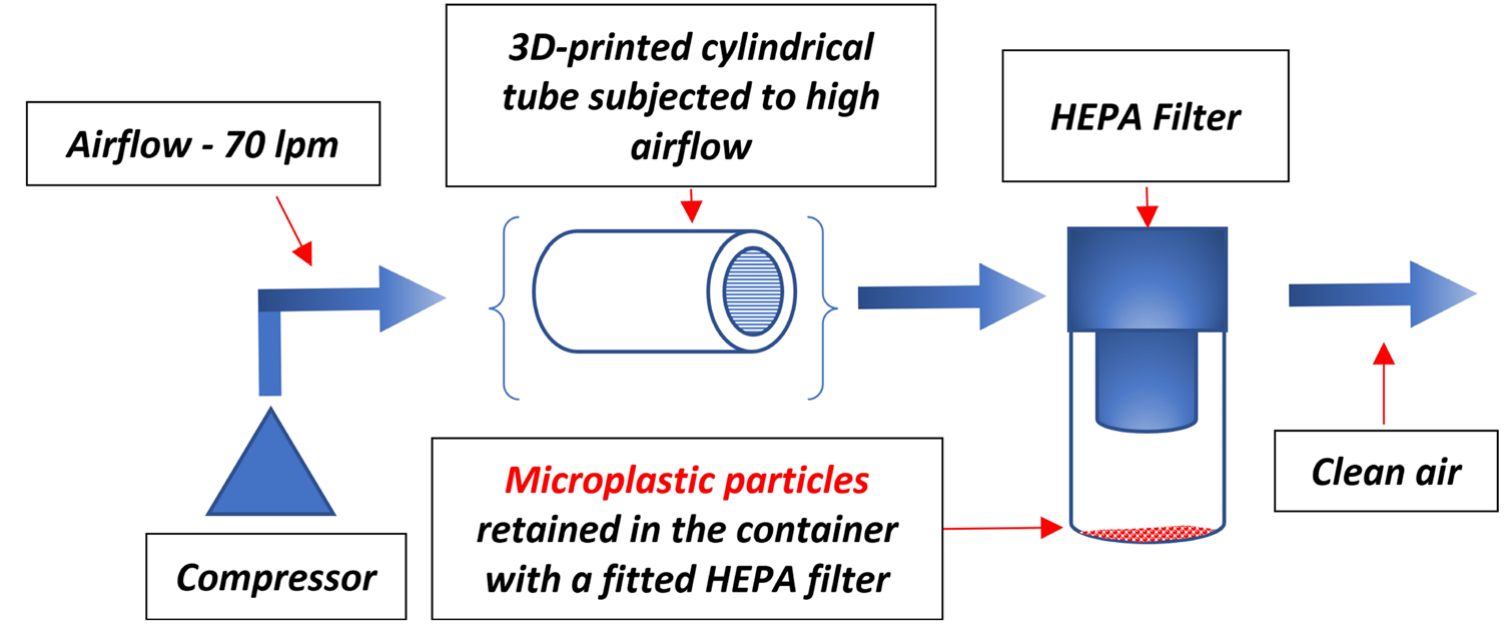
The laboratory stand was used to evaluate the risk of material particles being removed from the internal walls due to high airflow.

The laboratory set-up presented on the scheme (Fig. 12) was proposed to evaluate the erosive effects of high-flow air on the internal walls of 3D-printed components. Cylindrical test samples were printed using PET-G and ABS Medical filaments, with the ABS group further divided into two subtypes: printed with and without active cooling. Each sample measured 100 mm in length and had an internal diameter of 5.6 mm, corresponding to typical dimensions of respiratory tubes. Compressed air at an operating pressure of 0.02 MPa was continuously passed through the samples for a minimum of 6 h. Downstream of each sample, a membrane filter capable of capturing plastic particles larger than 40 µm was installed to collect any detached material. After airflow exposure, filters were carefully removed and analyzed using a Keyence VHX-6000 digital microscope equipped with automated particle recognition software. This system allowed for the precise detection and size of microparticles, with a detection resolution of approximately 1 µm.

**Fig. 13:**
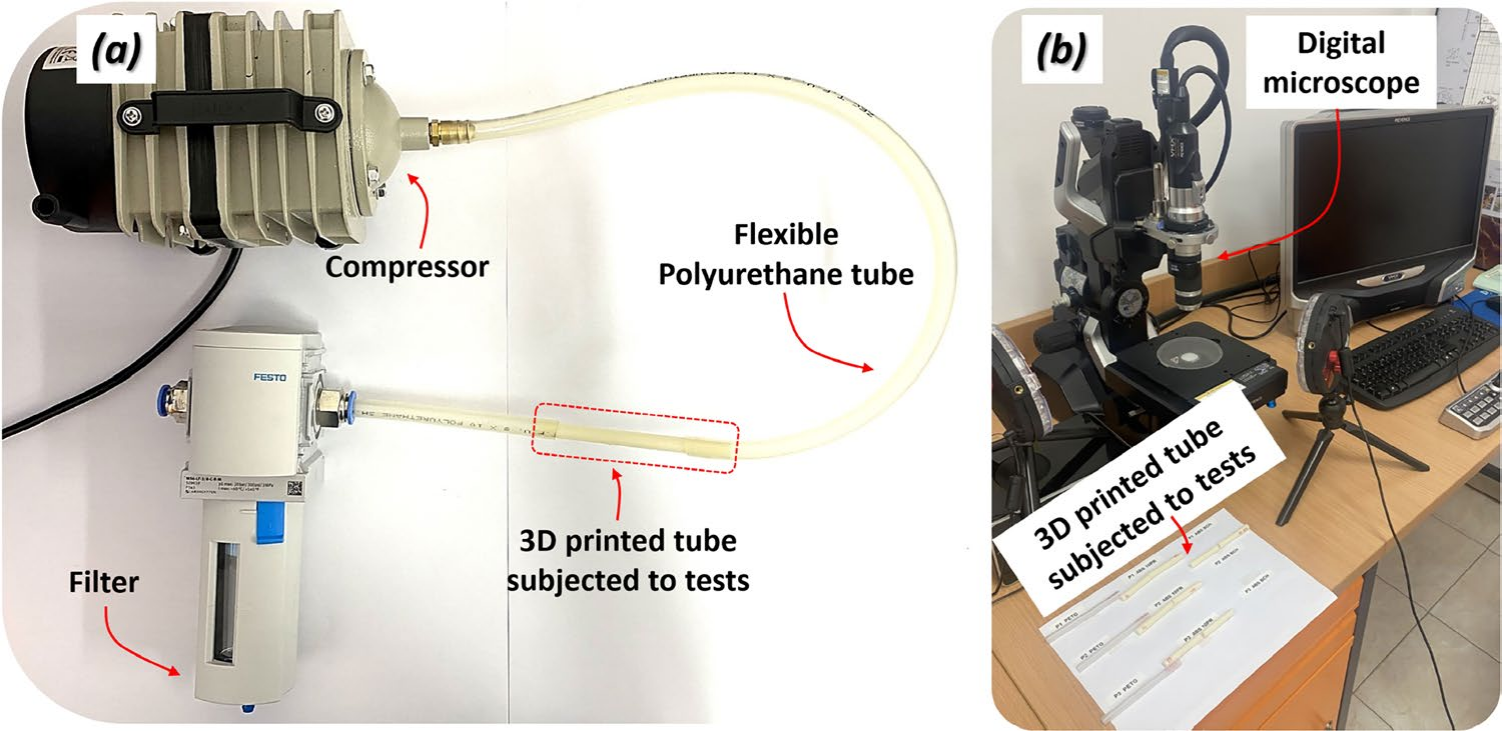
The main view of the laboratory stand applied to control the potential erosion of FFF 3D printed material subjected to high airflow (**a**) – set-up used to perform tests, (**b**) digital microscope to analyze gathered material particles.

The analysis included visual inspection of internal sample surfaces and post-test quantitative evaluation of the number, morphology, and size range of retained plastic particles. The main view of the laboratory setup used is presented in Fig. 13.

Surface quality optimization was performed using rectangular test samples printed from ABS Medical, TPU-95, and PET-G FX120 under varying FFF process parameters. The surface morphology of each specimen was evaluated using a Keyence VHX-6000 digital microscope, and only parameter sets that produced low visual roughness and high dimensional consistency were selected for the final part production. The methodology focused on evaluating particle detachment from tubes printed with PET-G and ABS Medical filaments, with the latter tested in two variants: with and without print cooling. The analysis included a preliminary microscopic inspection of the internal surfaces, followed by a post-test quantitative and qualitative assessment of particles captured by the filter.

Helmet inlet and outlet connectors were redesigned for 3D printing, using two methods: FFF and SLS. Due to the fine polymer powder (50–70 μm) used in SLS and the associated risk of unmelted particles detaching^39–41^ into the components of the helmet chamber, parts of the inlet connector (Fig. 4) were assigned to the FFF 3D printing method. Compared with SLS, FFF is less hazardous because it poses a lower risk of loose particles detaching from internal surfaces under high volumetric airflow rates.

For production, TPU 95 PolyFlex (PolyMaker, China) was chosen for T 22F-type sockets. ABS Medical and PET-G FX 120 (both from Spectrum Filaments, Poland) were selected for diffuser parts and the inlet connector with drain. TPU-95 supports vibration welding with the helmet, has a Shore hardness of 95A, high elasticity that allows for elongation three times, and a tensile strength of 29 ± 2.8 MPa. These properties enable a secure press fit with the breathing tube used in respiratory therapy.

Meanwhile, ABS Medical is characterized by biocompatibility in accordance with the USP Class VI and ISO 10,993-1 standards, allowing up to 30 days of contact with the human body, as well as compliance with EU Regulation No. 10/2011 and FDA 21 CFR regarding food contact. The material ensures high print quality, enabling the rapid production of durable components with strong interlayer adhesion. It exhibits thermal resistance with a Vicat softening temperature of 97° C and excellent mechanical properties, including a tensile strength of 47 MPa and elongation at break of 16%. It is ideal for producing customized prosthetics and structures that support rehabilitation (https://sklep.spectrumfilaments.com/product-pol-1257-Filament-Spectrum-ABS-Medical-1-75mm-1kg.html).

PET-G FX 120 is specifically designed for medical applications and offers high durability and strength. A key feature of this filament is its temperature resistance to 120 °C, allowing finished elements to undergo high-temperature steam sterilization. This makes it suitable for the manufacture of components related to prosthetics, orthoses, and other medical devices (https://sklep.spectrumfilaments.com/product-pol-1259-Filament-Spectrum-PET-G-FX120-1-75mm-NATURAL-1kg.html).

Before fabricating the inlet and outlet connectors, additional tests were performed to evaluate the erosive effects of high airflow (70 l/min) on 3D printed elements made from ABS Medical and PET-G. The aim was to assess whether these components could pose a health risk to hospitalized patients by shedding material particles into the helmet interior. Due to the potential for unmelted powder detachment, fittings produced using the SLS method, which is suitable only for external parts of the air supply system—were excluded from the study.

### Evaluation of the patients comfort and effectiveness of redesigned hCPAP

**Fig. 14:**
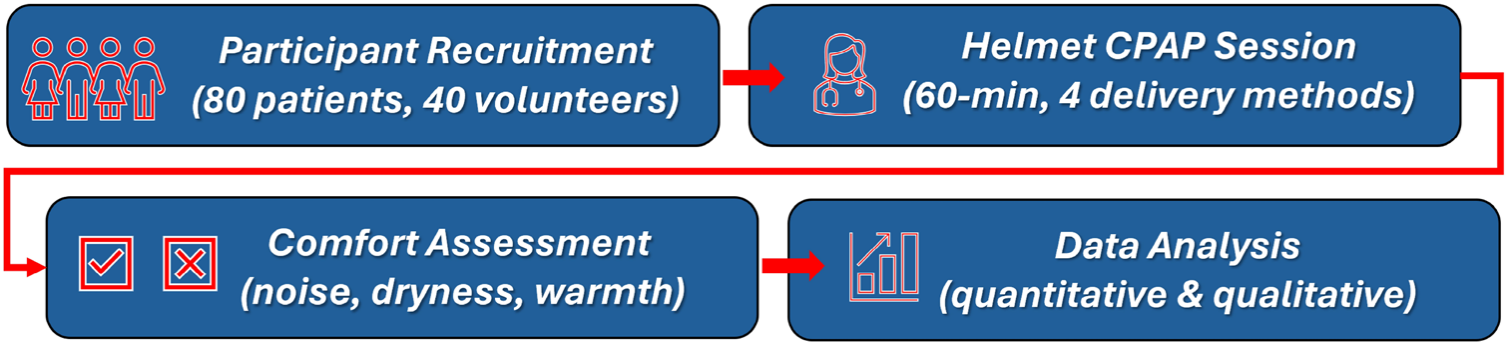
The scheme illustrating the main step of carried out medical trials carried out to evaluate the comfort of redesigned hCPAP.

To evaluate the ergonomic performance and therapeutic effectiveness of the redesigned helmet CPAP system, a structured clinical study was conducted involving both patients and healthy volunteers (Fig. 14). The trial included 120 participants in total, comprising 80 patients diagnosed with respiratory failure and 40 healthy individuals serving as controls. Each subject underwent a 60-min session of helmet CPAP therapy using one of four oxygen delivery configurations. The assessment focused primarily on patient comfort and perceived treatment conditions. Quantitative evaluation of noise perception was performed using a 10-point Likert scale, where 1 indicated the absence of noise and 10 corresponded to intolerable noise. In addition, qualitative feedback regarding dryness and thermal sensation (warmth) inside the helmet was collected through post-session questionnaires (Table 2).

**Table 2:** Key questions from the questionnaire used to assess participant comfort during oxygen therapy.

Category	Question / Statement	Response scale
Noise perception	Please rate the level of noise you experienced during the CPAP session	10-point Likert scale (1 = no noise, 10 = intolerable noise)
Dryness	Did you experience any dryness in your mouth, nose, or throat during the session?	5-point scale (1 = none, 5 = very severe)
Warmth	How would you rate the sensation of warmth inside the helmet?	5-point scale (1 = very comfortable, 5 = very uncomfortable)
Breathing comfort	How easy was it for you to breathe with the helmet during the session?	5-point scale (1 = very easy, 5 = very difficult)
General comfort	Overall, how comfortable did you feel during the session?	5-point scale (1 = very comfortable, 5 = very uncomfortable)
Additional remarks	Please provide any additional comments regarding your experience with the helmet system	Open-ended

All tests were conducted under consistent environmental conditions, with ambient temperature maintained between 22 and 24 °C and relative humidity between 40 and 50%. All medical tests were conducted according to ethical guidelines and approved by the Warsaw Bioethics Committee of the Medical University (approval No. 177/2020).

## Results

### Risk assessment of particle detachment in 3D printed components

**Fig. 15:**
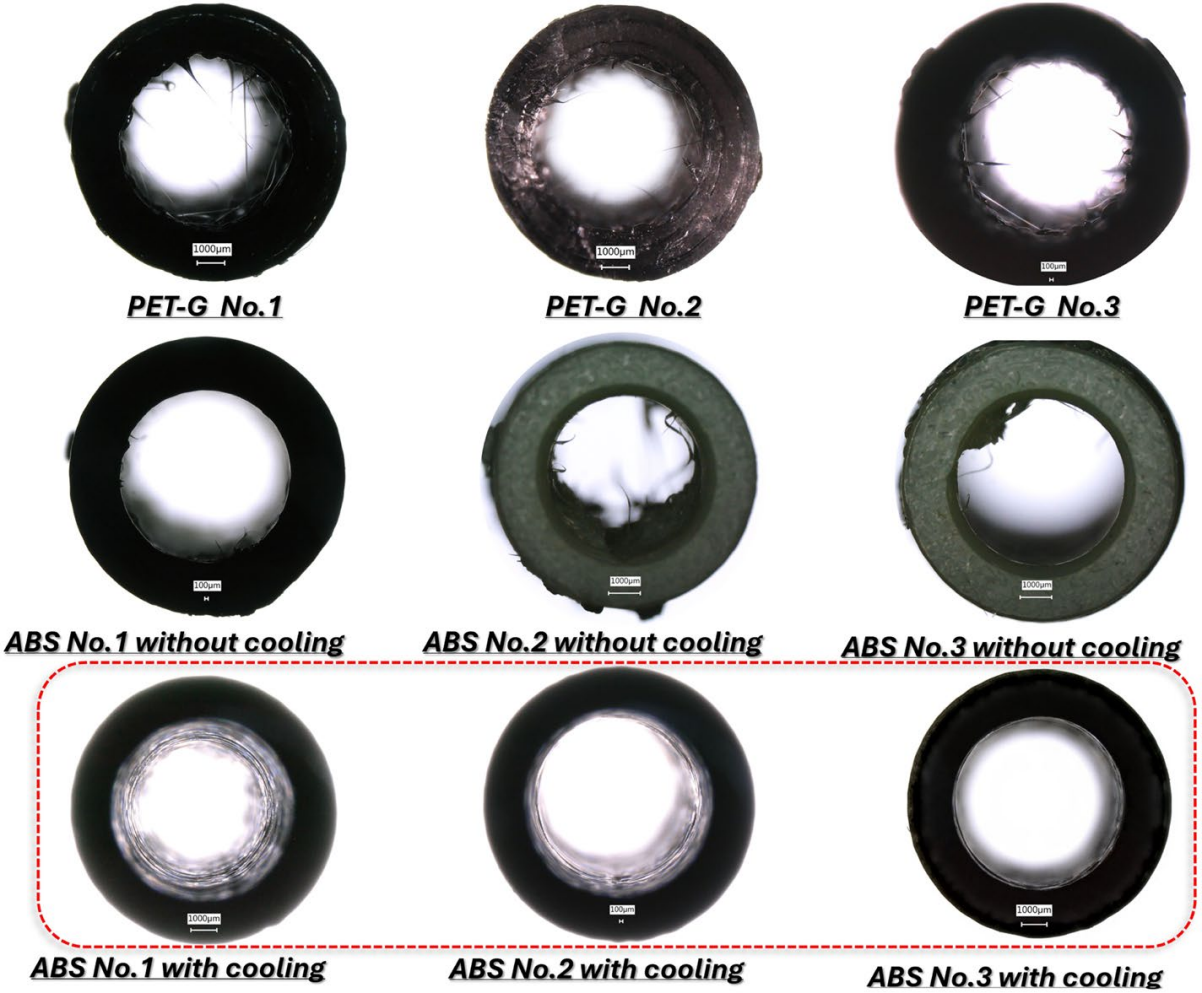
Registered microscope images of tube-shaped specimens made from PET-G and ABS medical filaments.

All three types of samples were subjected to airflow erosion testing: PET-G, ABS printed with cooling, and ABS printed without cooling (see Fig. 14). However, quantitative results are shown only for PET-G material in Fig. 15 and Table 3, because no microparticles larger than 40 µm were detected in either of the ABS variants. The filters used in the tests remained visually and analytically clean after both ABS tests, providing no data points to report. Each variant of material was tested on three replicates (n = 3) produced under identical printing conditions to ensure reproducibility. The presence and dimensions of any detached particles were evaluated using a Keyence VHX-6000 digital microscope equipped with automated particle recognition software, capable of detecting and measuring features down to 1 µm. The particle dimensions were recorded directly from the digital output without manual editing. This method ensured a consistent and quantitative approach to assessing surface erosion due to high-flow air.

**Table 3:** Results of the observations of microplastics retained in the filter housing after tests.

Sample number	Number of microplastic elements	Minimal size [µm]	Maximum size [µm]
PET-G No.1	24	57 × 93	535 × 194
PET-G No.2	20	41 × 41	358 × 437
PET-G No.3	10	51 × 57	207 × 181

Regarding the visual surface imperfections observed on ABS samples — commonly described as ‘hairs’, no correlation was found between their presence and the occurrence of detached microparticles. In particular, while ABS printed without cooling showed some loosely shaped surface structures, these remained bonded during the test and did not erode under forced airflow. On the contrary, the PET-G samples exhibited measurable erosion with particle detachment, which varied between samples despite identical print settings. This variation is considered to be an effect of the natural randomness of material erosion and small microstructural differences even in a single print batch.

The goal was to determine which material generated fewer microplastic particles due to erosive wear from airflow (Fig. 14). Samples were visually examined using a Keyence VHX-6000 digital microscope (KEYENCE Corporation, Osaka, Japan). The tubes were positioned to allow illumination and observation. As shown in Fig. 15, PET-G samples showed persistent, irregular, hair-like structures, even after adjusting printing parameters. A similar issue was observed in ABS Medical samples produced with the default (cooling enabled) settings. However, the disabling of the cooling option during ABS printing eliminated these structures, making this configuration the most favourable in terms of minimizing microplastic formation under high-flow conditions.

**Fig. 16:**
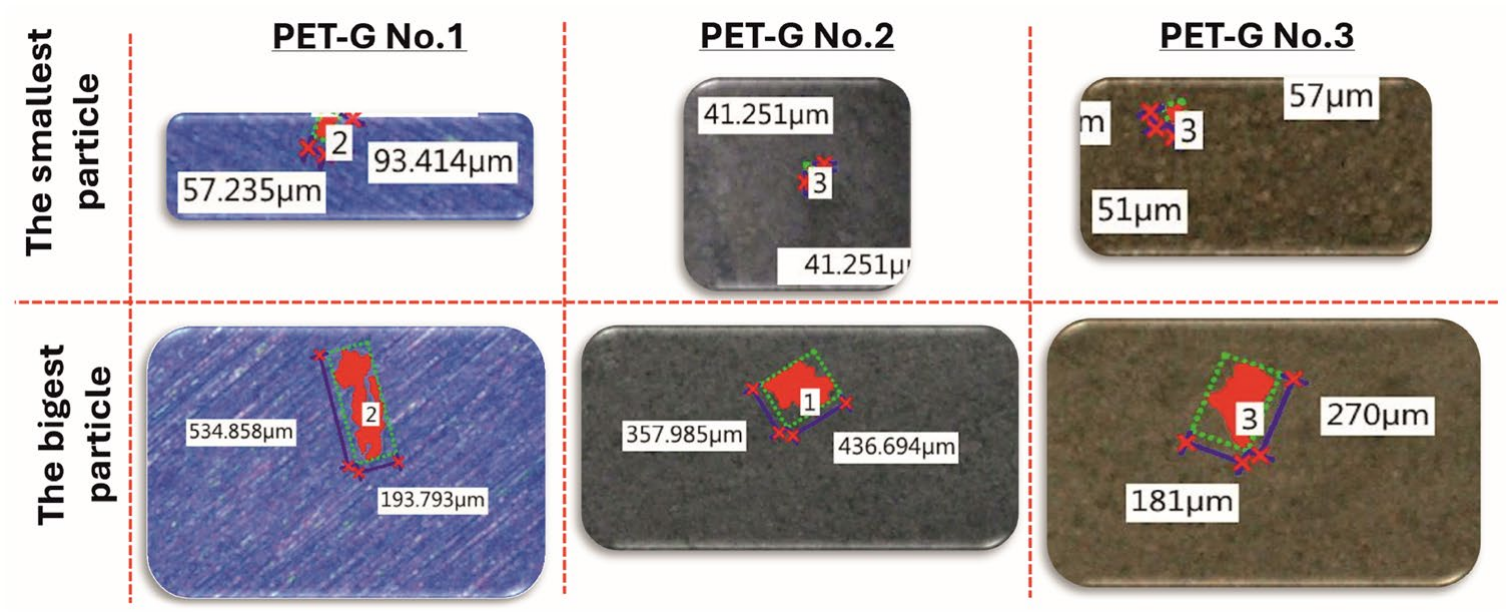
Microplastic size (the smallest and the largest) determined using the Keyence 6000 VHX digital microscope.

The next stage of verification of the potential detachment of plastic particles involved assessing the presence of microplastics in the housing with a HEPA filter used for testing. The material retained within the housing was placed on a laboratory glass slide, allowing the evaluation of the the number and size of particles using a Keyence 6000 VHX (KEYENCE Corporation, Osaka, Japan). Figure 16 illustrates images of the plastic particles captured by the filter. Furthermore, in Table 3, detailed measurements results are presented. Based on the assessment of their size, it was determined that the components of the inlet connector and diffuser would be manufactured using ABS Medical filament, with cooling disabled during the 3D printing process. In the case of tests conducted using samples made of ABS Medical material, no presence of microplastics was found in the HEPA filter housing. In addition to laboratory-scale evaluation of material integrity, the redesigned diffuser underwent a preliminary clinical evaluation to verify its ergonomic performance.

### Manufacturing process of modified variants of inlet and outlet connectors using 3D printing techniques

The inlet and outlet connectors of the hCPAP helmet were manufactured using additive manufacturing techniques, including Fused Filament Fabrication (FFF) and Selective Laser Sintering (SLS). T22F-type sockets were manufactured using a TPU-95 polyflex filament due to its flexibility, biocompatibility, and compatibility with vibration welding to the helmet shell. Other components, such as inlet connectors and diffusers, were produced from ABS Medical, selected for its low risk of microplastic release and stable mechanical performance under high airflow. The PEEP valves were printed using SLS with PA2200 polyamide powder, given their position on the exhalation path with no direct airflow toward the patient. Although initial attempts were made to fabricate the PEEP valve using the FFF technique. This approach is feasible; however, it requires support material, which lengthens the build time and adds post-processing steps to remove the supports. In view of this, we chose to manufacture the structural components of the PIP valve using selective laser sintering (SLS). SLS makes it possible to produce a large number of parts in a single build, thereby substantially reducing the time needed to complete the task. Additionally, the Authors made attempts to manufacture the PEEP valve components with an integrated spring to control piston preload. These attempts did not prove successful. Printed springs exhibited inconsistent preload under varying humidity and temperature conditions, and the coarse thread pitch limited the adjustability of the valve.

The geometries of all components were optimized for efficient 3D printing without support structures, significantly reducing fabrication time and material waste. A dedicated multi-printer setup enabled small-batch production, facilitating iterative design improvement and rapid prototyping under emergency response conditions. Each batch was subjected to post-processing and quality control to ensure functional reliability. Detailed 3D printing parameters, material properties, and slicer screenshots are provided in the Supplementary Materials (Appendix A).

Before final production, a series of iterative prototyping trials were conducted using rectangular and cylindrical specimens to optimize the process parameters. The primary objectives were to minimize surface roughness and evaluate dimensional deviations caused by thermal shrinkage, particularly in ABS medical material. Optimized results were achieved by disabling active cooling during extrusion, which significantly improved layer adhesion and geometric stability. Each sample was measured using a digital caliper (accuracy ± 0.01 mm), with dimensional deviations from nominal CAD values consistently below ± 0.25 mm for critical fit features. Surface quality was assessed both visually and using profilometry. The selected printing parameters, derived from these tests, ensured dimensional consistency and eliminated the need for post-processing. The final values used for all functional components are summarized in Appendix A, along with the mechanical properties of the applied materials.

A total of 150 helmet assemblies were produced using the described 3D printing process. Before deployment in clinical studies, all 3D-printed components were inspected to verify conformity with design tolerances and structural integrity. The validated assemblies were then provided for clinical evaluation described in Sect. “Results of the patients comfort and effectiveness of redesigned hCPAP”.

### Results of the patients comfort and effectiveness of redesigned hCPAP

To assess the ergonomic performance of the redesigned hCPAP helmet, a clinical evaluation was carried out in human trials. All procedures complied with ethical requirements and were approved by the Warsaw Bioethics Committee of the Medical University (approval No. 177/2020). Clinical use proceeded with informed consent. The assembled systems were used primarily in patients with non-COVID respiratory failure, with a subset deployed for emergency COVID-19 management during periods of peak demand.

The evaluation focused on short-term usability and comfort during 60-min CPAP sessions. Four oxygen-delivery methods were used according to clinical needs. Ambient conditions were kept constant (22–24 °C; 40–50% relative humidity). The primary endpoint was perceived noise, rated on a 10-point Likert scale (1 = no noise; 10 = intolerable). Secondary qualitative feedback on dryness and warmth was collected using a brief post-session questionnaire. In parallel, operational reliability was observed at the bedside.

A total of 120 individuals participated (n = 80 patients with respiratory failure; n = 40 healthy volunteers). No mechanical failures, leaks or flow disturbances attributable to 3D-printed artefacts were observed, and no adverse events related to the additively manufactured components were reported during clinical operation. Among patients, at a constant flow of 30 L min⁻^1^, the average perceived noise score was < 3/10. Volunteers reported similarly low noise scores across the flows tested. Although the study did not include objective sound-pressure measurements, the consistently low ratings indicate that the redesigned diffuser and flow path were effective in managing perceived noise during typical clinical use. Qualitative comments did not reveal systematic concerns about dryness or warmth under the stated conditions.

Taken together, these findings support the clinical usability of the redesigned helmet, particularly with respect to comfort and bedside reliability. The results suggest that the geometric optimisation of the diffuser and connectors helps to maintain low perceived noise while preserving functional performance. As this was a short-term, pragmatic evaluation centred on comfort and immediate usability, longer-term ergonomics, objective acoustic measurements and broader multi-centre testing are planned to substantiate and extend these observations.

The study confirms the clinical usability of the redesigned hCPAP system and supports the applicability of additive manufacturing for safe and ergonomic respiratory equipment. Future work will include long-term clinical trials and objective acoustic analyses to validate these initial results.

## Discussion

The implementation of additive manufacturing (AM) in the development of CPAP helmet connectors and diffusers demonstrates significant advantages over conventional manufacturing approaches, particularly in situations of disrupted supply chains such as the COVID-19 pandemic. Using FFF and SLS technologies, we were able to rapidly design, prototype, and produce functional components without the need for traditional injection moulding or tooling. This approach drastically reduced lead time and cost while maintaining design control and performance. Compared to commercial CPAP interfaces, which are typically standardized and inflexible, the 3D printed components developed in this study enabled customization on demand. The geometry of the connectors and diffusers was easily adjusted to fit different helmet shells, flow generators, or patient-specific needs. As noted in^16^ the use of PET-G and the application of Fused Filament Fabrication allowed Polish hospitals to locally produce hCPAP systems during critical supply shortages, ensuring continuity of care despite global logistical breakdowns. Additionally, the redesigned diffuser in our system contributed to a significant reduction in perceived noise levels, enhancing patient comfort. The ability to iteratively improve geometry through rapid prototyping aligns with the observations made by Zainul Arefeen et al.^39^, who emphasized the unique value of AM in enabling functional, patient-specific modifications under time constraints.

In a broader context, initiatives such as the 3D COVID Project in Paris (François et al.^40^) clearly demonstrated the strategic potential of 3D printing during health crises. Within four days, an entire production hub was deployed in a repurposed abbey, operating over 60 printers and supplying thousands of contact-free devices to public hospitals. This example highlights how AM enables rapid scaling of distributed manufacturing, eliminating the delays associated with tooling and procurement while responding directly to frontline needs.

The broader implication of this approach is its applicability in crisis scenarios. During the COVID-19 pandemic, 3D printing was widely adopted to manufacture personal protective equipment (PPE), respiratory connectors and even ventilator parts^16^,^16^ . Similarly, in military or humanitarian contexts, AM can enable point of care or field-deployable production of vital components^39^. With biocompatible, sterilizable polymers now widely available, the barrier to clinical implementation is lower than ever. In summary, our work validates the role of 3D printing as a versatile and resilient manufacturing method for medical applications. Beyond the immediate needs of the COVID-19 crisis, the proposed approach offers a blueprint for decentralized, adaptable healthcare manufacturing in future pandemics, conflicts, or supply chain disruptions. A key consideration in Additive Manufacturing of medical connectors is achievable dimensional tolerance, which directly affects press fit reliability and airtightness. Unlike traditional machining, where precise fit is ensured through tight tolerances and surface finishes, AM components often require design adaptation or compensatory materials. In our case, the use of flexible TPU allowed for dimensional compliance and leak-tight press-fit connections despite the inherent variability of FFF processes. This demonstrates the feasibility of functionally robust tool-free connectors tailored to AM capabilities.

This study has several limitations. Our conclusions are bounded by the manufacturing routes and equipment we used. The FFF prototypes were printed on open-frame Prusa i3 MK3S + with a standard slicer workflow and without an actively heated enclosure. Thermal control and airflow on this platform influence bead morphology, inter-layer bonding and surface finish, all of which can affect the presence of fine surface irregularities inside channels. Results obtained here should not be assumed to carry over to enclosed, industrial-grade FFF systems that use different thermal management, filtration or validated clean-manufacturing procedures. We also worked with a narrow set of filaments: PET-G, ABS-Medical and TPU-95A PolyFlex. These materials differ in rheology and surface energy, which shape stringing behaviour and the ease of producing smooth internal passages; the findings should not be generalised to other polymers or medical-grade formulations without direct testing. In addition, we did not examine how repeated cleaning or sterilisation, nor post-processing methods such as vapour or solvent smoothing, might change surface integrity or particle shedding.

Our stance on SLS was deliberately cautious. We did not carry out a full qualification of PA 2200 (PA12) produced on an EOS Formiga P110, and we did not quantify residual-powder release from enclosed geometries under clinically relevant airflow. The practical challenge of reliably depowdering narrow, tortuous channels remains central for components in the breathing-gas pathway. A dedicated follow-up is needed to measure powder retention and release (e.g., flush and airflow aerosolisation with particle counting), to assess the effects of powder ageing and refresh ratios, to verify depowdering protocols, and to evaluate cleanliness and biocompatibility after cleaning/sterilisation—ideally with reference to requirements such as ISO 18,562. Until those data are available, conclusions about the suitability of SLS for inlet-assembly parts should be treated as provisional.

Finally, the methodological scope was intentionally pragmatic. Airflow analysis combined bench measurements with steady-state CFD under simplified boundary conditions (rigid walls, idealised turbulence), which do not capture patient-specific breathing patterns or transient effects and may underestimate shear-driven particle mobilisation. The clinical component consisted of 60-min comfort sessions at a single centre; it provides useful early ergonomics information but no long-term data or evidence of clinical effectiveness. Future work will broaden printer classes (including enclosed, medical-oriented FFF systems), extend the range of materials, and, crucially, undertake the PA 2200/Formiga P110 programme outlined above to establish robust powder-removal, cleanliness and biocompatibility for respiratory applications.

## Conclusions

The presented case study on optimizing the geometry of the inlet and outlet fitting elements of the hCPAP helmet exemplifies a structured approach applicable to the design of novel medical equipment. The process was characterized by the integration of advanced computational design tools and state-of-the-art manufacturing techniques, particularly 3D printing. Furthermore, the methodology outlined in this study reflects the iterative nature of contemporary product development, emphasizing continuous improvement through successive design refinements.

Taking advantage of this foundational knowledge, the first concept design of the CPAP helmet was developed as a rapid response to the urgent need for respiratory support equipment during the early stages of the COVID-19 pandemic in 2019. Given the shortage of medical devices to treat acute respiratory failure, the proposed solution played a crucial role in addressing this pressing health challenge.

Subsequent clinical trials, initially involving volunteers and later expanding to patient participants, provided critical information on the strengths and limitations of the preliminary design. The findings of these trials were fundamental in the next phase of development, which focused on addressing identified shortcomings, optimizing the dimensions and weight, and simplifying the manufacturing process to improve overall efficiency and scalability. To summarize, the key modifications implemented by the authors to enhance patient comfort include:

- Eliminating the proposed Boston connector significantly reduced the size of both the inlet and outlet fittings. In addition, it considerably simplified the process of attaching the connectors to the helmet.
- Integration of F-22 sockets, manufactured using advanced 3D printing techniques, designed in compliance with the relevant EN ISO 5356-1:2015 standard, and subsequently bonded to CPAP helmets via vibration welding. This approach ensured a robust and reliable connection while leveraging the benefits of additive manufacturing.
- Significant reduction in overall dimensions and weight of both inner and outer connectors by replacing the conventional threaded joint with a press-fit connection. This change contributed to improved portability and user comfort while maintaining structural integrity.
- Optimization of connector geometry through CAD-based modifications, carefully considering the constraints and capabilities of widely available 3D printing technologies, specifically Fused Filament Fabrication (FFF) and Selective Laser Sintering (SLS). This refinement enhanced manufacturability, ensuring precision and consistency in component production.
- Additional tests have demonstrated that PET-G, despite its high technological adaptability and suitability for manufacturing components intended for food contact or skin exposure, is not suitable for the production of components used in oxygen therapy. This limitation arises due to the formation of fine, hair-like structures during the 3D printing process, which are challenging to eliminate.
- In contrast, ABS Medical, although susceptible to thermal shrinkage, exhibits a lower tendency to develop such technological defects. Therefore, the authors recommend its use in the manufacture of medical device components, particularly connectors used in oxygen therapy, such as the CPAP helmet connectors characterized in this study.

The design and manufacturing approach presented in this study exemplifies an effective response to crisis scenarios, including pandemics, armed conflicts that lead to supply chain disruptions, and restricted access to essential medical treatments.

## Supplementary Information

Supplementary Information 1.

Supplementary Information 2.

Supplementary Information 3.

Supplementary Information 4.

Supplementary Information 5.

## Data Availability

The 3D CAD models developed by the Authors (in *.step and *.stl formats) can be made available to readers upon request directed to the Corresponding Author.
