# Supplementary material for: Design, manufacturing, and testing of 3D-printed fittings for ergonomic helmet CPAP devices: a case study
Source: Sci Rep. 2025 Nov 25;15:41870. doi: 10.1038/s41598-025-25851-2 (PMC12647709; doi:10.1038/s41598-025-25851-2)
Supplement: Supplementary file 1 — Supplementary Information 1. [file 41598_2025_25851_MOESM1_ESM.docx]

**Appendix A. Detailed 3D printing parameters, slicing settings and mechanical parameters of the used materials**

**Appendix A.1 - 3D printing parameters, slicing settings and mechanical parameters of the used materials**

Table A.1 FFF 3D Printing Parameters for TPU-95 PolyFlex components

| **Parameter** | **Minimal value** | **Maximum value** | **Identified value** |
| --- | --- | --- | --- |
| Layer thickness [mm] | 0.2 | 0.4 | 0.2 |
| Extrusion width [mm] | 0.4 | 0.5 | 0.4 |
| Nozzle temperature [°C] | 210 | 230 | 210 |
| Bed temperature [°C] | 25 | 60 | 50 |
| Cooling option [%] | 0 | 100 | 0 |
| 3D printing speed [mm/s] | 30 | 50 | 30 |
| Retraction length [mm] | 1 | 3 | 1 |
| Retraction speed [mm/s] | 35 | 60 | 35 |

Table A.2 Mechanical properties of 3D printed TPU-95 PolyFlex material

| Parameter | Value |
| --- | --- |
| Young’s Modulus [MPa] | 9.4 ± 0.3 |
| Tensile Strength [MPa] | 29.0 ± 2.8 |
| Elongation at break [%] | 330.1 ± 14.9 |
| Shore hardness | 95 A |
| Melting Temperature [°C] | 210 |
| Density [kg/m³] | 1200 |

Table A.3 FFF 3D Printing Parameters for ABS Medical components

| **Parameter** | **Minimal value** | **Maximum value** | **Identified value** |
| --- | --- | --- | --- |
| Layer thickness [mm] | 0.2 | 0.4 | 0.2 |
| Extrusion width [mm] | 0.4 | 0.5 | 0.45 |
| Nozzle temperature [°C] | 235 | 255 | 240 |
| Bed temperature [°C] | 100 | 100 | 100 |
| Cooling option [%] | 0 | 25 | 25 |
| 3D printing speed [mm/s] | 30 | 150 | 40 |
| Retraction length [mm] | 0.5 | 2 | 0.8 |
| Retraction speed [mm/s] | 25 | 45 | 40 |

Table A.4 Mechanical properties of 3D printed ABS Medical material

| **Parameter** | **Value** |
| --- | --- |
| Young’s Modulus [MPa] | 2450 |
| Yield stress [MPa] | 47 |
| Elongation at break [%] | 16 |
| Ball indentation hardness [N/mm^2^] | 120 |
| Vicat softening temperature [°C] | 97 |
| Density [kg/m³] | 1060 |

Table A.5 SLS 3D Printing Parameters for PA 2200 (PEEP Valve)

| **Parameter** | **Identified value** |
| --- | --- |
| Layer thickness [mm] | 0.1 |
| Contour speed [mm/s] | 3000 |
| Contour Power [W] | 13.5 |
| Contour beam offset [mm] | 0 |
| Hatching distance [mm] | 0.25 |
| Hatching speed [mm/s] | 3200 |
| Hatching Power [W] | 24 |
| Hatching beam offset [mm/s] | 0.12 |

Table A.6 Mechanical properties of PA 2200 material

| **Parameter** | **Value** |
| --- | --- |
| Young’s Modulus [MPa] | 1650 |
| Tensile Strength [MPa] | 48 |
| Elongation [%] | 18 |
| Melting Temperature [°C] | 176 |
| Softening Temperature (1.80 MPa) [°C] | 70 |
| Softening Temperature (0.65 MPa) | 154 |
| Density [kg/m³] | 930 |

**Appendix A.2 - Additional Notes on Manufacturing**

- All components were designed to be printable without support structures.
- Multi-printer setup: 6× Prusa i3 MK3S+ for parallel batch production were used
- Typical production times:

- T22F sockets: 4 per 4h batch

- Inlet connectors: 4 per 4h batch

- Diffusers: 18 in 3h batch

- PEEP valves (SLS): printed in a full build chamber using the Formiga P110 system (approx. 24 h print time); batch post-processing took approximately 2 hours.

Representative screenshots from PrusaSlicer, illustrating the slicing process and model layout for selected components (e.g., T22F socket, inlet connector, diffuser), are presented below to complement the described manufacturing parameters (Figure A.1÷A.4).


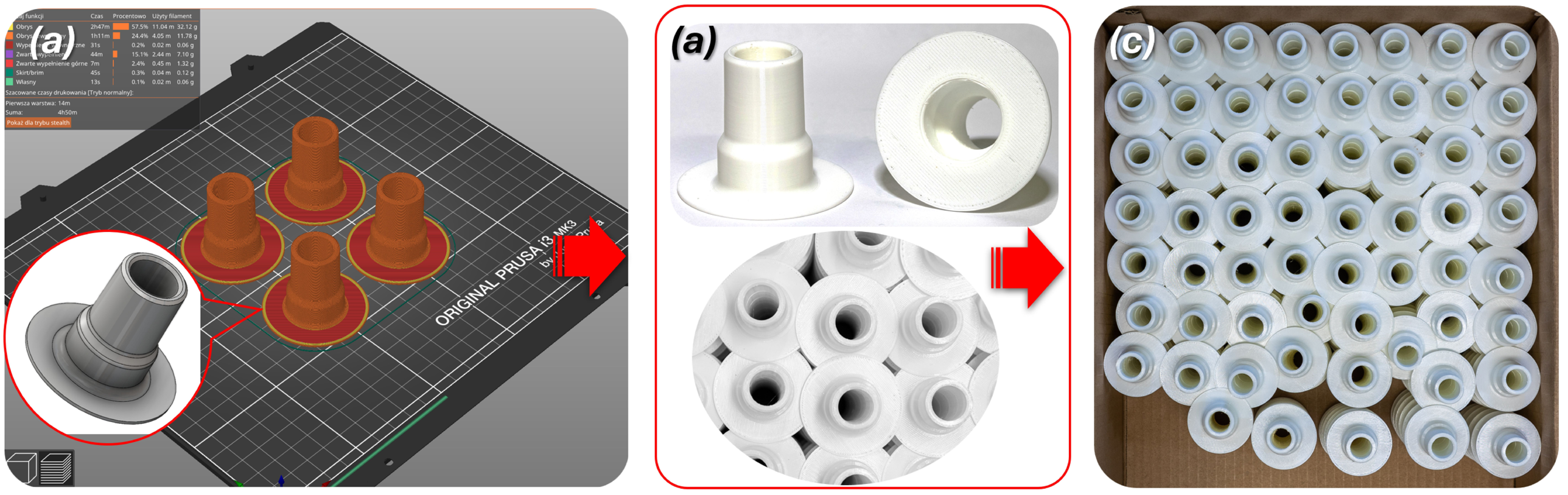


**Figure A.1.** Preparation and manufacturing of T22F connector sockets. (a) PrusaSlicer (FFF) workspace showing the 3D model and layer-by-layer slicing used to generate G-code; (b) examples of fabricated sockets - single part and close-up of a batch; (c) small-batch production - representative tray from the series (total n = 360).


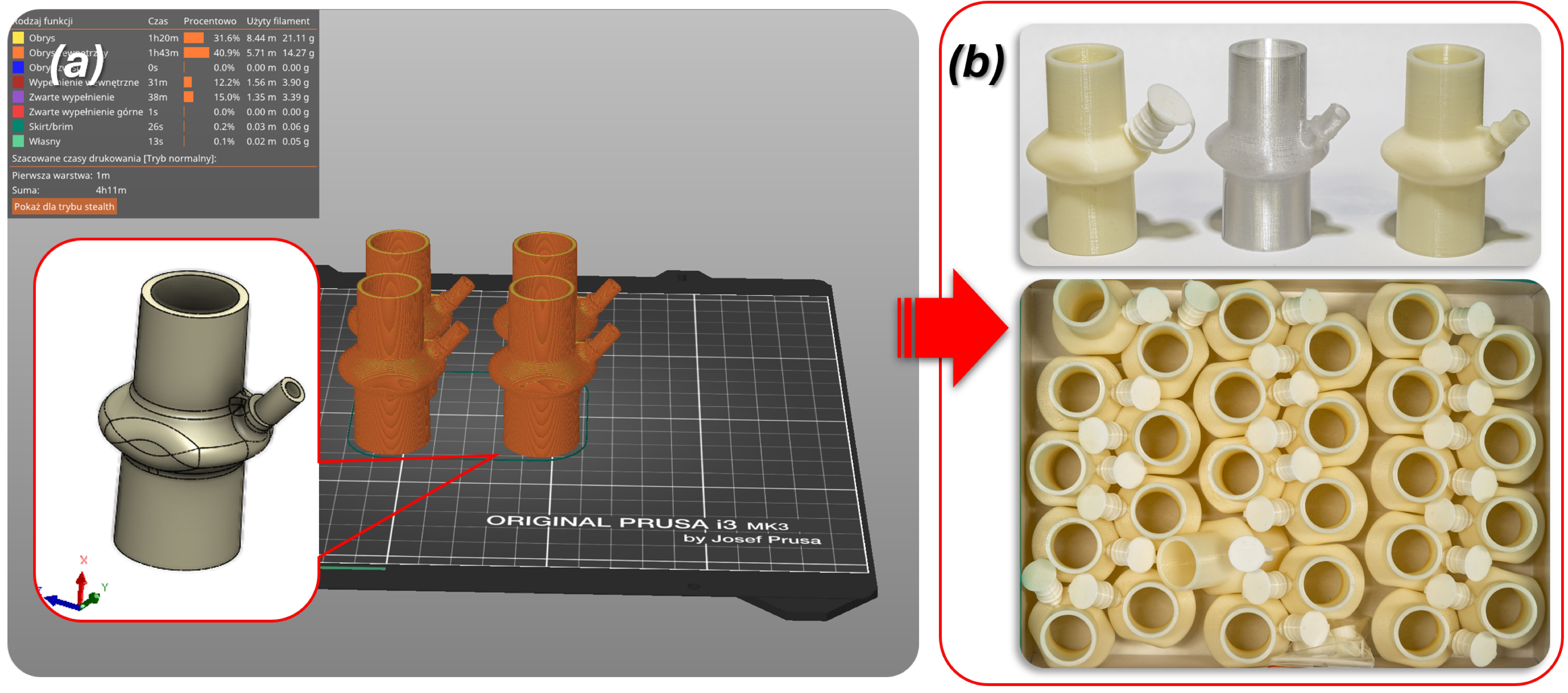


**Figure A.2.** Design and fabrication of the single-inlet connector with auxiliary oxygen port: (a) PrusaSlicer workspace showing the 3D model and layer-by-layer slicing used to generate the G-code; (b) additively manufactured connectors - detail of individual parts (top) and a produced batch (bottom).


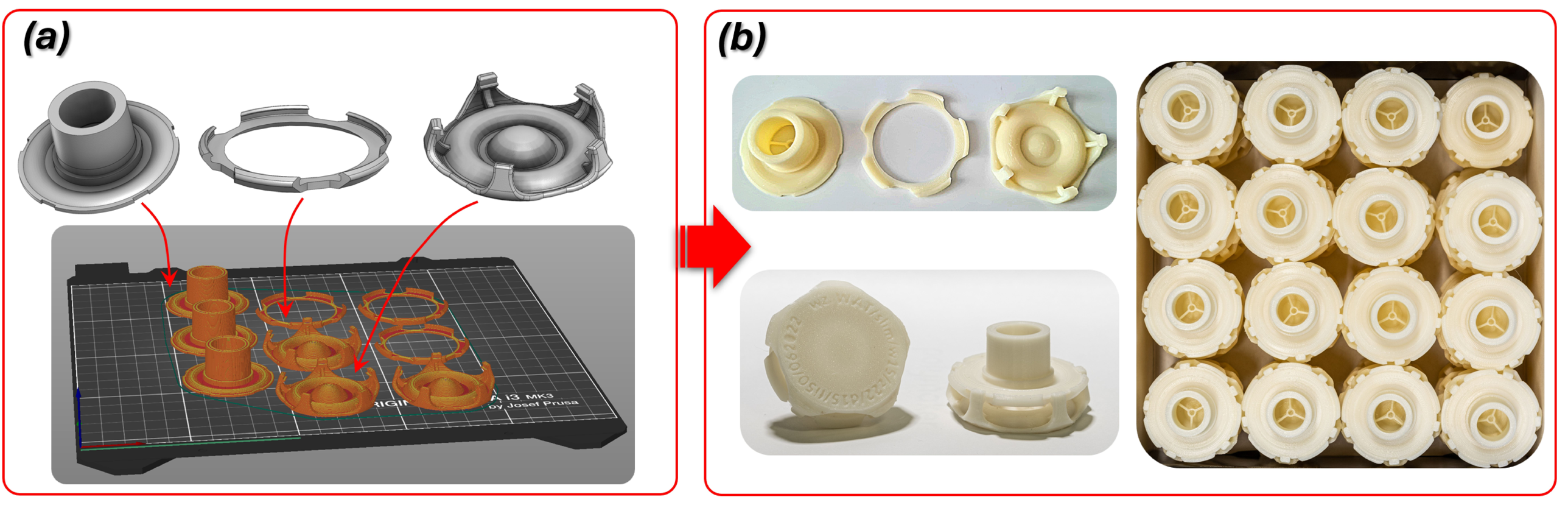


**Figure A.3.** Diffuser - design and FFF fabrication: (a) 3D models of the constituent parts with build layout and slicing preview in PrusaSlicer; (b) additively manufactured parts - detail of individual components and a representative production batch.


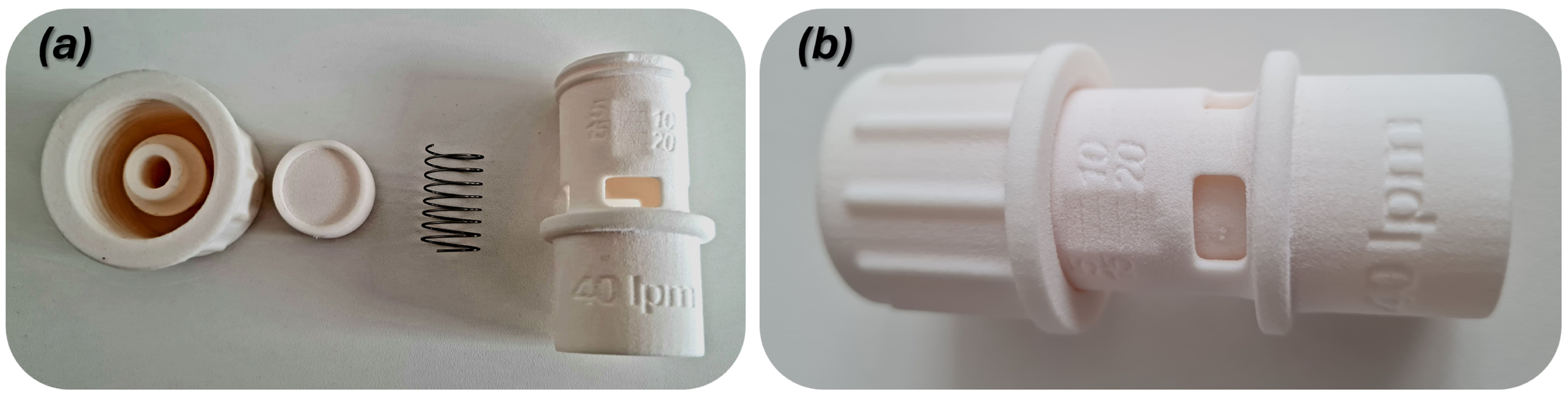


**Figure A.4.** Main view of the PEEP valve manufactured using the SLS technique:
(a) - view of individual components, (b) – view of assembled elements
