# Supplementary figures and images for: Design, manufacturing, and testing of 3D-printed fittings for ergonomic helmet CPAP devices: a case study
Source: Sci Rep. 2025 Nov 25;15:41870. doi: 10.1038/s41598-025-25851-2 (PMC12647709; doi:10.1038/s41598-025-25851-2)

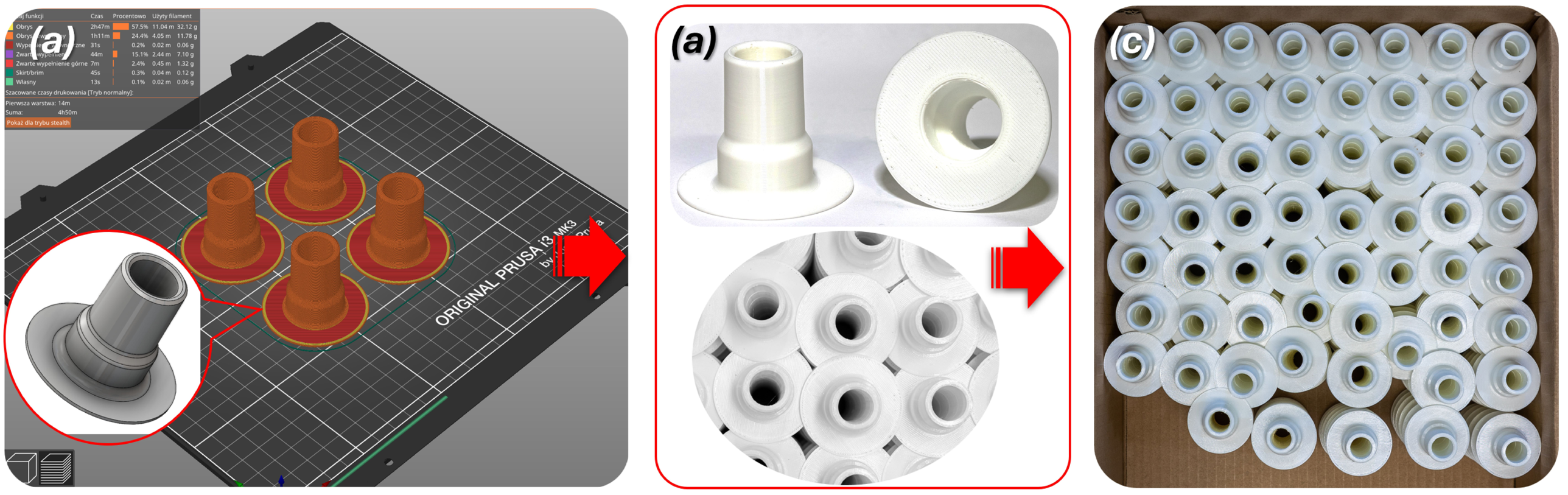

Supplement: Supplementary file 2 — Supplementary Information 2. [file 41598_2025_25851_MOESM2_ESM.jpg]

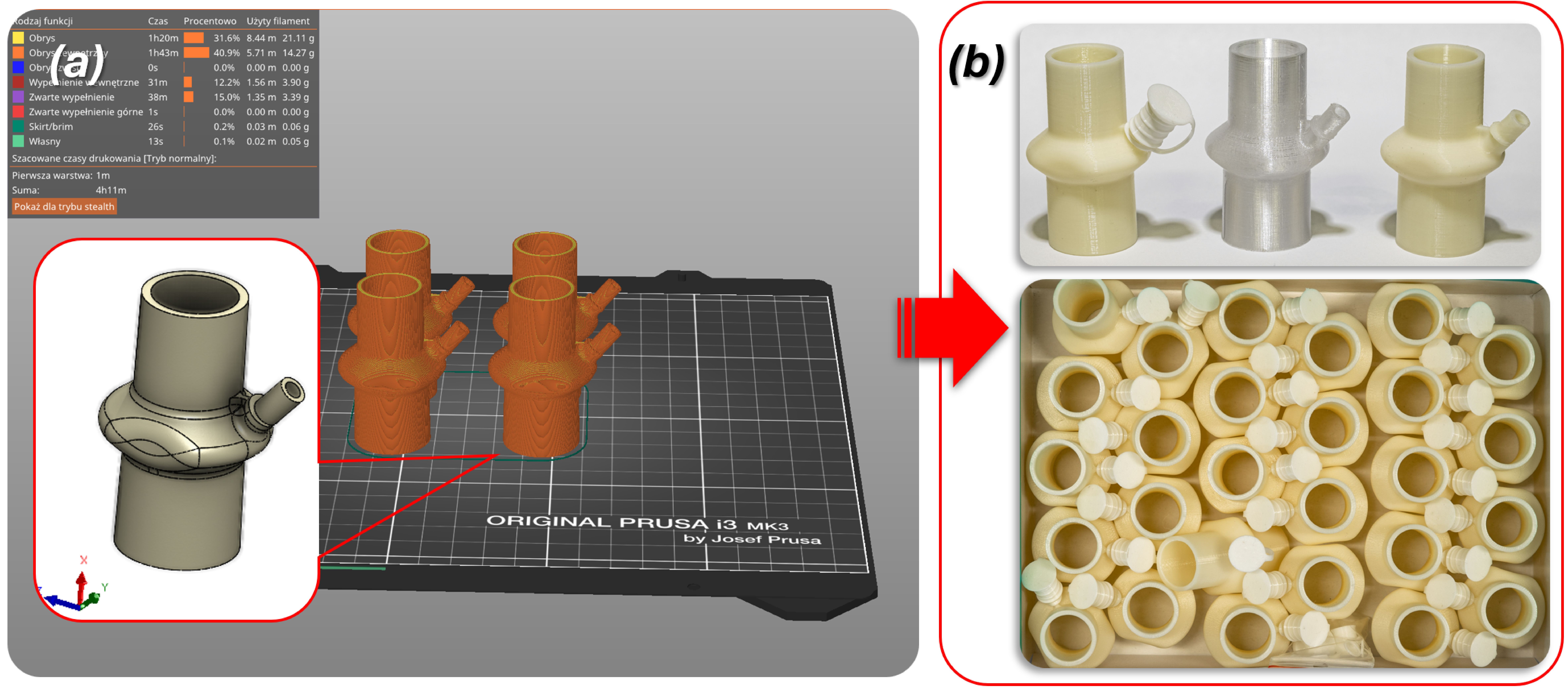

Supplement: Supplementary file 3 — Supplementary Information 3. [file 41598_2025_25851_MOESM3_ESM.jpg]

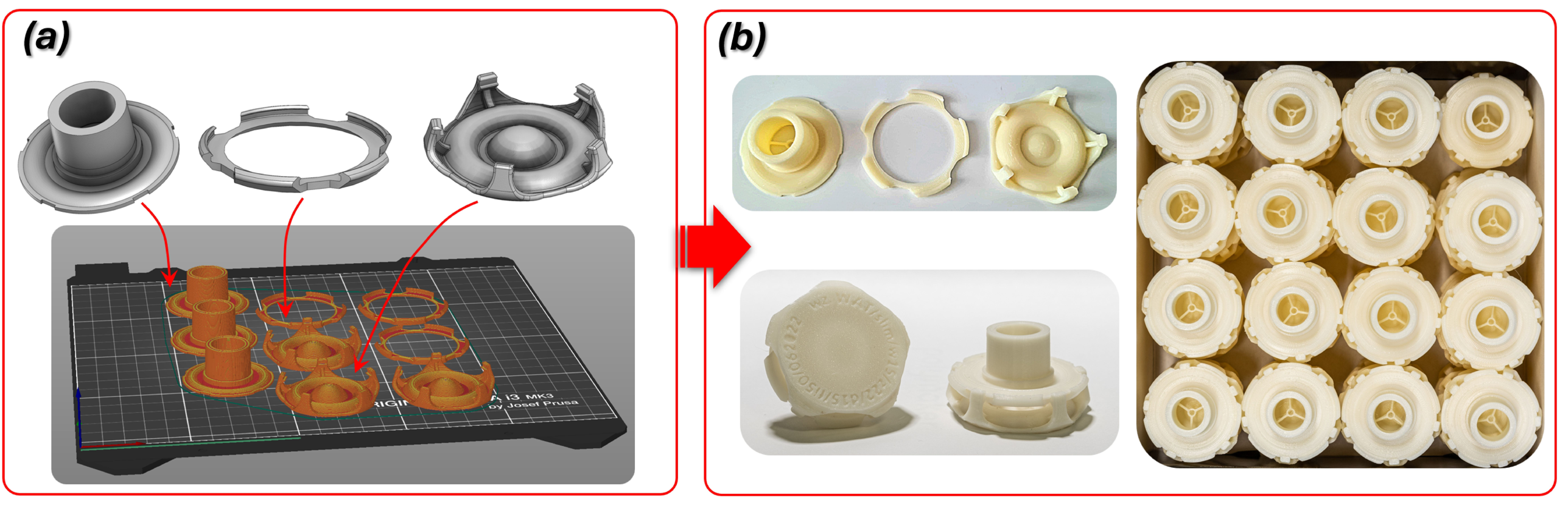

Supplement: Supplementary file 4 — Supplementary Information 4. [file 41598_2025_25851_MOESM4_ESM.jpg]

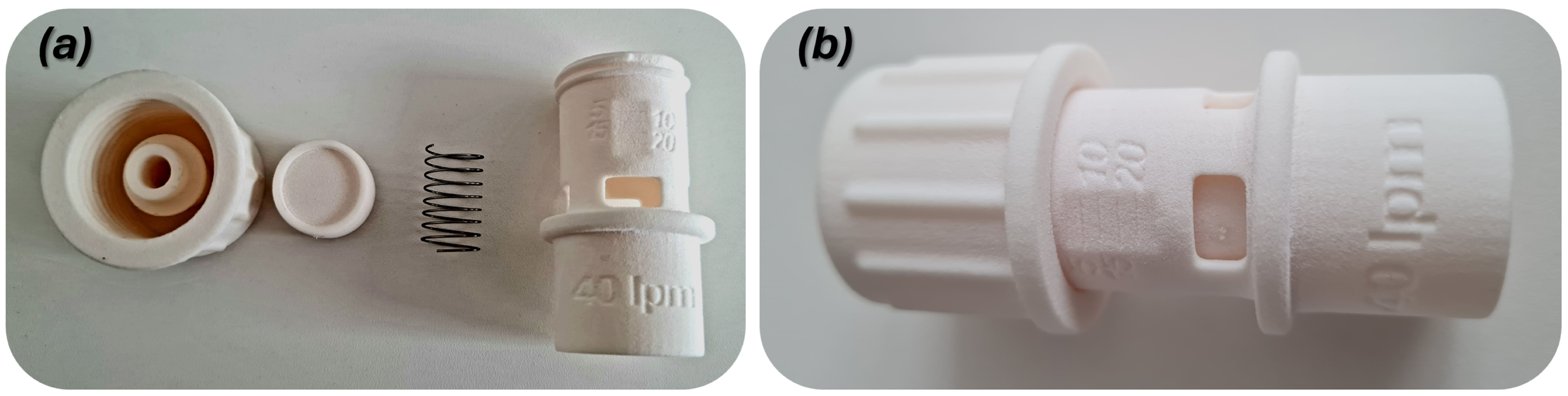

Supplement: Supplementary file 5 — Supplementary Information 5. [file 41598_2025_25851_MOESM5_ESM.jpg]
